# Supplementary material for: Pervasive cooperative mutational effects on multiple catalytic enzyme traits emerge via long-range conformational dynamics
Source: Nat Commun. 2021 Mar 12;12:1621. doi: 10.1038/s41467-021-21833-w (PMC7955134; doi:10.1038/s41467-021-21833-w)
Supplement: Supplementary file 1 — Supplementary Information [file 41467_2021_21833_MOESM1_ESM.pdf]

Supplementary Information

Pervasive cooperative mutational effects on multiple catalytic enzyme traits emerge via long-range conformational dynamics

Carlos G. Acevedo-Rocha[1], Aitao Li[2], Lorenzo D'Amore[3], Sabrina Hoebenreich[4], Joaquin Sanchis[5], Paul Lubrano[1,X], Matteo P. Ferla[6], Marc Garcia-Borràs[3], Sílvia Osuna[3,7]* and Manfred T. Reetz[4,8,9]*

[1] Biosyntia ApS, 2100 Copenhagen, Denmark

[2] State Key Laboratory of Biocatalysis and Enzyme Engineering, Hubei Collaborative Innovation Center for Green Transformation of Bio-Resources, Hubei Key Laboratory of Industrial Biotechnology, School of Life Sciences, Hubei University, Wuhan, 430062, P. R. China

[3] Institut de Química Computacional i Catàlisi and Departament de Química, Universitat de Girona, Carrer Maria Aurèlia Capmany 69, Girona 17003, Catalonia, Spain

[4] Department of Chemistry, Philipps-University Marburg, 35032, Germany

[5] Monash Institute of Pharmaceutical Sciences, Monash University, 399 Royal Parade, Parkville 3052, VIC, Australia

[6] Wellcome Centre for Human Genetics, University of Oxford, Roosevelt Drive, Oxford OX3 7BN, UK

[7] ICREA, Pg. Lluís Companys 23, 08010 Barcelona, Spain

[8] Department of Biocatalysis, Max-Planck-Institut für Kohlenforschung, Kaiser-Wilhelm-Platz 1, 45470 Mülheim an der Ruhr, Germany

[9] Tianjin Institute of Industrial Biotechnology, Chinese Academy of Sciences, 32 West 7th Avenue, Tianjin, 300308, P. R. China

[X] Current address: Bacterial Metabolomics group, Eberhard Karls Universität Tübingen, Geschwister-Scholl-Platz, 72074 Tübingen, Germany

These authors contributed equally to this work: Carlos G. Acevedo-Rocha, Aitao Li, Lorenzo D'Amore

*Corresponding authors: silvia.osuna@udg.edu and reetz@mpi-muelheim.mpg.de

Table of Contents

SUPPLEMENTARY NOTES **3**

Supplementary Note 1: Kinetics of purified enzymes. *3*
Supplementary Note 2: Quantum mechanical calculations. *3*
Supplementary Note 3: Molecular Dynamics simulations. *4*
Supplementary Note 4: Accelerated Molecular Dynamic Simulations. *5*
Supplementary Note 5: Additivity terms and equations. *6*
Supplementary Note 6: Automated calculation of additivity using Python 3.7. .... *8*
Supplementary Note 7: Pathway accessibility for multiple catalytic parameters towards 2β-hydroxytestosterone. *10*
Supplementary Note 8: Conformational population analysis. *11*
Supplementary Note 9: Shortest Path Map analysis. *11*
Supplementary Note 10: Additional Packages. *11*

SUPPLEMENTARY FIGURES **12**

Supplementary Figure 1: Catalytic mechanism for aliphatic C-H hydroxylation catalyzed by P450s. *12*
Supplementary Figure 2: SDS-PAGE analysis of purified mutants. *13*
Supplementary Figure 3: QM transition-states for the C–H abstraction in 1 from (a) C-2 (b) C-15. .... *13*
Supplementary Figure 4: Snapshot of 1 binding pose of during MD simulations in 15β-selective mutants. *14*
Supplementary Figure 5: Snapshot of binding pose during MD simulations in 2β-selective mutants. *14*
Supplementary Figure 6: KDE plot of the second replica dataset. *15*
Supplementary Figure 7: KDE plot of the third replica dataset. *15*
Supplementary Figure 8: PC Analysis (pc2) of the substrate-bound mutant (a) -II and (b) III. *16*
Supplementary Figure 9: Computed active site volume for mutants (a) III and (b) ---. *16*
Supplementary Figure 10: Final pose of 1 in the aMD binding trajectory of I-- *17*
Supplementary Figure 11: (1)CO-Y51 polar interaction interaction. *17*
Supplementary Figure 12: PC Analysis (pc2) of substrate free mutant (a) III and (b) -II. *18*
Supplementary Figure 13: RMSF of selected back-bone elements in mutants ---, -II and III. *18*
Supplementary Figure 14: Pathway accessibility for multiple enzymatic parameters towards 2β-hydroxytestosterone. *19*
Supplementary Figure 15: PC Analysis (pc2) of apo-state mutated enzymes along the fitness landscape pathways. *20*
Supplementary Figure 16: Conformational population analysis (pc1, pc2) of deconvoluted mutants. *21*

SUPPLEMENTARY TABLES **22**

Supplementary Table 1: Kinetic profiles of deconvoluted mutants. *22*
Supplementary Table 2: Energies, thermal corrections and free energies of the QM structures calculated for H-abstraction catalyzed by haem. *22*
Supplementary Table 3: Additivity calculations for substrate conversion to 2β-hydroxytestosterone. *23*
Supplementary Table 4: Additivity calculations for 2β-hydroxytestosterone selectivity. *23*
Supplementary Table 5: Additivity calculations for NADPH consumption rate (NCR) to 2β-hydroxytestosterone. *23*
Supplementary Table 6: Additivity calculations for product formation rate (PFR) to 2β-hydroxytestosterone. *23*
Supplementary Table 7: Additivity calculations for coupling efficiency (CE) towards 2β-hydroxytestosterone. *24*
Supplementary Table 8: Additivity calculations for TTN towards 2β-hydroxytestosterone. *24*
Supplementary Table 9: Additivity calculations for TTF towards 2β-hydroxytestosterone. *24*
Supplementary Table 10: Pathway accessibility analysis based on selectivity towards 2β-hydroxytestosterone (2). *25*
Supplementary Table 11: Pathway accessibility analysis based on TTF towards 2β-hydroxytestosterone (2). *25*
Supplementary Table 12: Oligonucleotides used in this study. *25*

SUPPLEMENTARY REFERENCES **25**

Supplementary Notes

Supplementary Note 1: Kinetics of purified enzymes.

Cytochrome P450s catalyze complex reactions involving many components such as substrate [S], product [P], cofactor (NADPH), and oxygen (O_2). These multicomponent enzymes exhibit atypical or so-called non-Michaelis Menten kinetics[1]. Steady state kinetics parameters (e.g., kcat/ Km) may lead to inaccurate interpretations on enzyme performance because these do not consider the effects of changing substrate concentration and product inhibition during the biocatalytic reaction.[2] Consequently, in order to assess the catalytic efficiency of the $P450_{BM3}$ variants, we determined the following parameters:

- Substrate (testosterone) conversion based on %HPLC.
- Selectivity (2β hydroxytestosterone) based on %HPLC (values converted to kJ per mol[3]).
- NADPH leak rate (NLR) is µmol NADPH consumed in the absence of substrate per µmol enzyme per min.
- NADPH consumption rate (NCR) is the total µmol NADPH consumed (to convert substrate into products) per µmol enzyme per min.
- Product formation rate (PFR) is determined by the multiplication of NCR with CE and has same units as NCR, namely, µmol products/NADPH per µmol enzyme per min.
- Coupling efficicency (CE) is the ratio between total consumption of substrate vs. NADPH and is expressed in %.
- Total turnover number (TTN) refers to total amount of products formed by enzyme concentration after NADPH depletion. The units are µmol product per µmol enzyme.
- Total turnover frequency (TTF) is calculated by dividing TTN values by total time (after NADPH depletion). The units are µmol product per µmol enzyme per min.

Supplementary Note 2: Quantum mechanical calculations.

The P450 protein-bound porphyrin complex coordinated with iron and containing an Fe=O moiety is known as compound I (Cpd I). In our computational model for Cpd I, substituents at the periphery of the porphyrin moiety were replaced with hydrogen atoms. Additionally, the axial Fe−SCys bond in Cpd I was modeled as a $Fe{-}SCH_3$ bond. Hydroxylation by P450 consists in the hydrogen atom abstraction from the C–H bond of the substrate by CpdI (which is the rate limiting step), followed by radical rebound mechanism. In agreement with previous work[4] the quartet C–H abstraction transition-states have been calculated to be consistently lower in energy than the doublet spin states. Hence, all the reported activation free-energies ($\Delta G^{\ddagger}$) correspond to C–H abstraction on the quartet potential energy surface.

A comprehensive computational DFT study was then performed with the Gaussian09(D.01)[5] program package using the B3LYP[6,7] hybrid functional under the unrestricted formalism (except for **1**, restricted singlet) and LANL2DZ pseudopotential with associated basis set[8] to describe the Fe atom and relativistic effects. All the other atoms were treated with a 6-31g(d) basis set during geometry optimization and subsequent frequency calculation, whereas 6-311(d,p) basis set was employed for final energies single point calculations. In all the calculations, solvent effects were taken into account using the Polarizable Continuum Model[9] (PCM) and dispersion energy correction was treated under the Grimme's D_3 formalism[10]. Supplementary Table 11

summarizes the energies, thermal corrections and free energies of the QM structures calculated for H-abstraction catalyzed by haem.

Supplementary Note 3: Molecular Dynamics simulations.

Long-timescale conventional Molecular Dynamics simulations (cMD) in explicit water were performed using AMBER 16 package[11] in our in-house GPU cluster Galatea. Substrate testosterone (**1**) parameters for the MD simulations were generated within the antechamber module of AMBER 16 using the general AMBER force field (GAFF),[12] with partial charges set to fit the electrostatic potential generated at the HF/6-31G(d) level by the restrained electrostatic potential (RESP) model.[13] The charges were calculated according to the Merz-Singh-Kollman scheme[14,15] using Gaussian 09(D.01).[5] Parameters for the haem resting state, Cpd I and axial Cys were taken from elsewere.[16] Amino acid protonation states were predicted using the H++ server (http://biophysics.cs.vt.edu/H++).[17] Then, the enzyme was solvated in a pre-equilibrated truncated hexagonal box with a 10-Å buffer of TIP3P[18] water molecules using the AMBER16 leap module, resulting in the addition of ~13,000 solvent molecules. The systems were neutralized by addition of explicit counterions (Na^+ and Cl^-). All subsequent calculations were done using the widely tested Lindorff-Larsen modification of the Amber 99 force field (ff99SBildn).[19] For all the mutants, the apo structures used were generated from the P450-BM3 original variant (PDB: 1FAG)[20], by removal of the palmitoleic acid bound to the protein and the introduction of the corresponding mutations using the RosettaBackrub web-server (https://kortemmeweb.ucsf.edu/backrub).[21–23] Substrate-bound structures were produced using the former mutants generated with RosettaBackrub as template, by placing the testosterone (**1**) molecule in the position originally occupied by the palmitoleic acid. Pose 2 and pose 15 were obtained by orienting **1** with its C2 or C15 atom towards the haem-moiety, respectively. The stability of all the aforementioned apo- and substrate-bound structures was tested by MD simulations. A two-stage geometry optimization approach was performed. The first stage minimizes the positions of solvent molecules and ions imposing positional restraints on solute by a harmonic potential with a force constant of 500 kcal mol^{-1} $Å^{-2}$, and the second stage is an unrestrained minimization of all the atoms in the simulation cell. The systems are gently heated using six 50-ps steps, incrementing the temperature 50 K each step (0–300 K) under constant volume and periodic boundary conditions. Water molecules were treated with the SHAKE algorithm such that the angle between the hydrogen atoms is kept fixed. Long-range electrostatic effects were modeled using the particle-mesh-Ewald method.[24] An 8-Å cutoff was applied to Lennard-Jones and electrostatic interactions. Harmonic restraints of 10 kcal/mol were applied to the solute, and the Langevin equilibration scheme was used to control and equalize the temperature. The time step was kept at 1 fs during the heating stages, allowing potential inhomogeneities to self-adjust. Each system was then equilibrated without restrains for 2 ns with a 2-fs timestep at a constant pressure of 1 atm and temperature of 300 K. After the systems were equilibrated in the NPT ensemble, for both apo and substrate-bound structures, three independent 600 ns MD simulations (i.e. 1.8 μs accumulated) were performed under the NVT ensemble and periodic-boundary conditions using our Galatea cluster (composed by 178 GTX1080 GPUs). With Galatea, simulations for these systems were performed at a speed of ca. 100 ns/day.

Supplementary Note 4: Accelerated Molecular Dynamic Simulations.

Long-timescale accelerated Molecular Dynamics simulations (aMD)[25–27] have been used to explore the binding trajectory of **1** in the different mutants. To allow the substrate to diffuse freely until being spontaneously recognized by the enzyme surface and finally getting in the access channel, we started with four different molecules of **1** randomly placed in the bulk solvent, performing three independent replicas, each one of 250 ns unconstrained cMD simulations (*vide supra*), from which the acceleration parameters were determined, followed by 750 ns of dual-boost aMD.

aMD enhances the conformational sampling by adding a bias potential (i.e. boost potential), which raises energy minima while keeping high energy regions almost unaffected. This smoothens the free energy landscape and enhances the sampling of rather slow conformational exchanges. In particular, a non-negative boost potential is added to the system when the system potential is lower than a reference energy:

$$V^*(r) = V(r), \qquad V(r) \geq E, \qquad (1)$$

$$V^*(r) = V(r) + \Delta V(r), \qquad V(r) < E \qquad (2)$$

where $V(r)$ is the original potential, E is the reference energy, and $V^*(r)$ is the modified potential. In the simplest form, the boost potential, $\Delta V(r)$ is given by:

$$\Delta V(r) = \frac{[E - V(r)]^2}{\alpha + E - V(r)} \qquad (3)$$

where α is the acceleration factor. As the acceleration factor α decreases, the energy surface is flattened more and biomolecular transitions between the low-energy states are increased. Here a total boost potential is applied to all atoms in the system in addition to a more aggressive dihedral boost, i.e., (E_{dihed}, α_{dihed}; E_{total}, α_{total}), within the dual-boost aMD approach. The acceleration parameters used in this work, are the following:

$$E_{\mathrm{dihed}} = V_{\mathrm{dihed\_avg}} + 2.0 \times N_{\mathrm{res},}\ \alpha_{\mathrm{dihed}} = 2.0 \times \mathrm{N_{res}}/5_{,} \qquad (4)$$

$$E_{\mathrm{total}} = V_{\mathrm{total\_avg}} + 0.16 \times N_{\mathrm{atoms},}\ \alpha_{\mathrm{total}} = 0.16 \times \mathrm{N_{atoms}} \qquad (5)$$

where N_{res} is the number of protein residues, N_{atoms} is the total number of atoms, and V_{dihed_avg} and V_{total_avg} are the average dihedral and total potential energies calculated from 250 ns cMD simulations, respectively.

Supplementary Note 5: Additivity terms and equations.

Mathematically, the addition between A and B can be determined according to the trait in quest.

In the case of stereoselectivity or enantioselectivty[3], the free energy of interaction ($\Delta G^{\neq}_{AB}$) between two mutations (or sets of mutations) *A* and *B* can be calculated using equation (6):

$$\Delta \mathrm{G}^{\neq}_{AB} = \Delta\Delta \mathrm{G}^{\neq}_{exp} - (\Delta\Delta \mathrm{G}^{\neq}_{A} + \Delta\Delta \mathrm{G}^{\neq}_{B}) \qquad (6)$$

Where $\Delta\Delta \mathrm{G}^{\neq}_{exp}$ is the difference in activation energy with respect to regioselectivity (**2** versus **3** formation and all the rest of the stereo- and regioisomers) experimentally obtained for the binary combination, while $\Delta\Delta \mathrm{G}^{\neq}_{A}$ and $\Delta\Delta \mathrm{G}^{\neq}_{B}$ are the experimental energies obtained for each mutant (or sets of mutations) independently.

In the case of conversion (or any other protein trait that cannot be used in equation 6 above that is logarithmic for enantioselectivity or regioselectivity), herein we introduce equation (7):

$$\Delta \mathrm{C}^{\%}_{AB} = \Delta \mathrm{C}^{\%}_{exp} - \left(\Delta \mathrm{C}^{\%}_{A} + \Delta \mathrm{C}^{\%}_{B}\right) \qquad (7)$$

Where $\Delta \mathrm{C}^{\%}_{exp}$ is the difference in conversion for **2** and the parent mutant F87A experimentally obtained for the binary combination, while $\Delta\% C_A$ and $\Delta\% C_B$ are the differences in the experimental conversion values between each mutant (or sets of mutations) separately and that of the parent mutant F87A (28 ± 1%): The conversion values are not logarithmic (in contrast to the selectivity ones) and thus the value of the parental mutant F87A has to be subtracted from the single-point mutants and the double mutant.

In any case, additivity for selectivity (conversion) occurs when $\Delta \mathrm{G}^{\neq}_{AB} = 0\ (\Delta \mathrm{C}^{\%}_{AB} = 0)$, while positive (+ME) and negative (-ME) magnitude epistasis correspondingly pertains when $\Delta \mathrm{G}^{\neq}_{AB} > 0\ (\Delta \mathrm{C}^{\%}_{AB} > 0)$ and $\Delta \mathrm{G}^{\neq}_{AB} < 0\ (\Delta \mathrm{C}^{\%}_{AB} < 0)$. Positive (+SE) and negative (-SE) sign epistasis occurs when $\Delta \mathrm{G}^{\neq}_{AB} > 0\ (\Delta \mathrm{C}^{\%}_{AB} > 0)$ if $A < 0\ or\ B < 0$ and $\Delta \mathrm{G}^{\neq}_{AB} < 0\ (\Delta \mathrm{C}^{\%}_{AB} < 0)$ if $A > 0\ or\ B > 0$, respectively. Finally, positive (+RSE) and negative (-RSE) reciprocal sign epistasis occurs when $\Delta \mathrm{G}^{\neq}_{AB} > 0\ (\Delta \mathrm{C}^{\%}_{AB} > 0)$ if $A < 0\ and\ B < 0$ or $\Delta \mathrm{G}^{\neq}_{AB} < 0\ (\Delta \mathrm{C}^{\%}_{AB} < 0)$ if $A > 0\ and\ B > 0$, respectively[28].

To exemplify the various cases of additivity/non-addivity, if the desired fitness trait is **2-beta hydroxytestosterone selectivity**, the following theoretical examples are given:

1. **Additivity:** Mutant A is 70% 2-beta sel. (2.1 kJ/mol). Mutant B is 79% 2-beta sel. (3.3 kJ/mol). Mutant AB is 90% 2-beta sel. (5.4 kJ/mol).

2. **Positive Magnitue Epistasis (+ME):** Mutant A is 70% 2-beta sel. (2.1 kJ/mol). Mutant B is 63% 2-beta sel. (1.3 kJ/mol). Mutant AB is 90% 2-beta sel. (5.4 kJ/mol).

3. **Positive Sign Epistasis (+SE):** Mutant A is 70% 2-beta sel. (2.1 kJ/mol). Mutant B is 63% 15-beta sel. (-1.3 kJ/mol). Mutant AB is 90% 2-beta sel. (5.4 kJ/mol).

4. **Positive Reciprocal Sign Epistasis (+RSE):** Mutant A is 70% 15-beta sel. (-2.1 kJ/mol). Mutant B is 63% 15-beta sel. (-1.3 kJ/mol). Mutant AB is 90% 2-beta sel. (5.4 kJ/mol).

5. **Negative Magnitue Epistasis (-ME):** Mutant A is 70% 2-beta sel. (2.1 kJ/mol). Mutant B is 63% 2-beta sel. (1.3 kJ/mol). Mutant AB is 60% 2-beta sel. (1.0 kJ/mol).

6. **Negative Sign Epistasis (-SE):** Mutant A is 70% 2-beta sel. (2.1 kJ/mol). Mutant B is 63% 15-beta sel. (-1.3 kJ/mol). Mutant AB is 90% 15-beta sel. (-5.4 kJ/mol).

7. **Negative Reciprocal Sign Epistasis (-RSE):** Mutant A is 70% 2-beta sel. (2.1 kJ/mol). Mutant B is 63% 2-beta sel. (1.3 kJ/mol). Mutant AB is 90% 15-beta sel. (-5.4 kJ/mol).

Using equations (6) and (7), the degree of interaction (if any) between two mutations (or sets of mutations) can be calculated.

All the calculations for the double mutants were performed as exemplified above for the binary combination of two single mutations (A+B) for **2** (Entry 1-6, Supplementary Tables 3-9). The additivity analyses were also performed for the ternary (i+j+k) combination of **2** (Entry 7, Supplementary Tables 3-9).

Supplementary Note 6: Automated calculation of additivity using Python 3.7.

The present study yielded a consequent amount of data with different equations (Supplementary Note 5). It was decided to automatize the data analysis process to gain time and reliability. To do so, a python 3.7 code was written with the Spyder interface. The code is deposited as https://github.com/matteoferla/Epistasis_Calculator and is divided in two parts:

The first part requires the user's input on different variables. The user indicates the number of mutations and the names. The user also needs to choose the right algorithm. There are two algorithms: Selectivity (S) or Conversion (C), which will have an impact for the final output of the program. The latter can be used for any parameter, except enantioselectivity. The output (excel tables) names also need to be given.

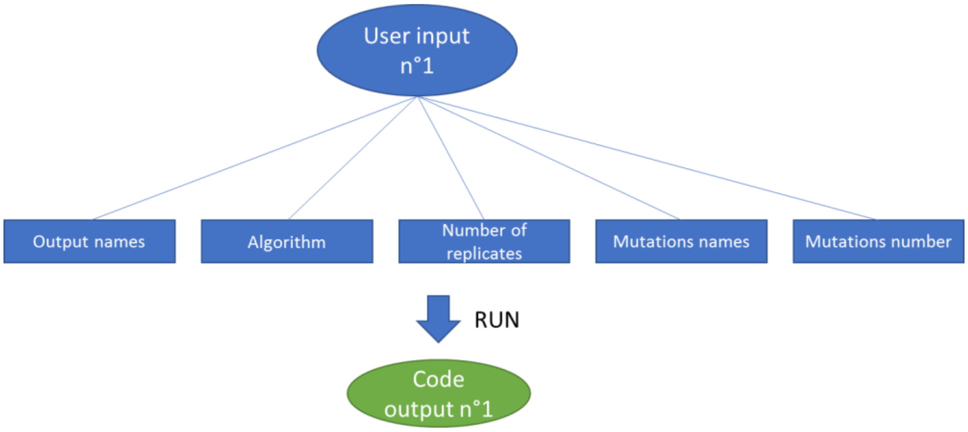

	Mutation 1	Mutation 2	Mutation 3	Replicate n°1	Replicate n°2	Replicate n°3
Mutant 1	-	-	-	X	X	X
Mutant 2	-	-	+	X	X	X
Mutant 3	-	+	-	X	X	X
Mutant 4	+	-	-	X	X	X
Mutant 5	+	+	-	X	X	X
Mutant 6	+	-	+	X	X	X
Mutant 7	-	+	+	X	X	X
Mutant 8	+	+	+	X	X	X

In the figure above, the user is asked to enter various variables. The code uses the variables to build its first output. In this table, the user will be able to enter data and proceed. In the example above, three mutations and three replicates were used. Their names were not specified for clarity. "X" filled cells are emplacement that the user needs to put its data to proceed.

The program uses all these variables to build its first output which is a mutant table. The mutants are represented with signs "+" and "-" for each mutation. A "+" means that the mutant has the associated mutation. The program uses a randomized algorithm to generates all the possible mutants. Each time a new mutant is assembled, it is introduced in the table and if it is already present, the program discards it. The number of mutants is related to the number of mutations. For three mutations, eight mutants can be obtained, sixteen for four mutations. Because of the randomization, the first output varies for every use.

Once the user has entered the data in the first output, the program is used again on this table to yield the second final output. Using the different mutants, the program creates different combinations of mutants to obtain other mutants. For example, mutation 5 above is the result of the combination of mutations 3 and 4. Mutant 8 can be obtained by using either three different

mutant pair combinations (6 and 3, 5 and 2, or 7 and 4) or a combination of three single mutations (2, 3 and 4). For a three mutations analysis, the number of combinations is seven, but it reaches thirty-six combinations when using four mutations.

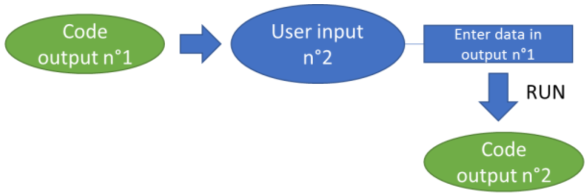

	Mutation 1	Mutation 2	Mutation 3	Combinations	Experimental average	Experimental standard deviation	Thoretical average	Theoretical standard deviation	Exp.avg - Theor.avg	Epistasis type
Combination n°1	-	+	+	(2, 3)	79,444162	2,411977329	33,80111833	1,122567494	45,64304367	"+ SE"
Combination n°2	+	+	-	(3, 4)	29,74640567	0,575299405	40,11042533	1,8107601	-10,36401967	"- SE"
Combination n°3	+	-	+	(2, 4)	26,51919833	1,337062731	45,412603	1,130403143	-18,89340467	"- ME"
Combination n°4	+	+	+	(2, 5)	68,412413	4,422365667	30,40450067	2,025752938	38,00791233	"+ SE"
Combination n°5	+	+	+	(4, 7)	68,412413	4,422365667	86,411564	3,449793811	-17,999151	"- ME"
Combination n°6	+	+	+	(3, 6)	68,412413	4,422365667	21,87511567	2,009929373	46,53729733	"+ RSE"
Combination n°7	+	+	+	(2, 3, 4)	68,412413	4,422365667	40,76852033	0,213766362	27,64389267	"+ SE"

In the second part of the program, the code assembles different combinations and attribute them different values based on the user's values. The program uses equations (6) or (7) given above (Supplementary Note 5) to give the results: Epistasis type. + = positive; - = negative; SE = "Sign Epistasis"; "ME" = Magnitude epistasis"; "RSE" = "Reciprocal Sign Epistasis".

After assembling the combinations, the program retrieves the values of their associated mutants and builds up "experimental" values. Each mutant of the first output is being given an average and standard deviation corresponding to the user's values. The program then assembles these values to yield "theoretical values". How the program uses these values is dictated by the "Selectivity" or "Conversion" algorithm. By comparing the theoretical values to the experimental ones, the program obtains a "score" (see Exp.avg – Theor.avg in previous figure). This score is what defines the nature of the epistatic interaction based on the equations (1) and (2) and the program gives its result. The final output is a table containing theoretical and experimental addition as well as type and degree of epistatic interaction.

The server can be used to calculate any fitness value for any combination in protein evolution. In the server, various examples (demos) are added to illustrate this feature. However, it should be noted that most studies use different equations for determining epistatic effects.

- Minimum inhibitory concentration (MIC) in µg/mL for β-lactamase towards cefotaxime[29].
- MIC in µg/mL for metallo-β-lactamase towards cephalexin[30].
- Catalytic efficiency in kcat/Km for dihydrofolate reductase towards trimethoprim[31].
- Melting temperature (Tm) of transketolase in °C[32].
- Enantiomer excess in kJ/mol for phenyl acetone monooxygenase towards *R*-sulfoxide[33].

Finally, there is also one example of a partial deconvolution:

- Catalytic efficiency in kcat/Km for formate dehydrogenase towards NADPH[34].

Supplementary Note 7: Pathway accessibility for multiple catalytic parameters towards 2β-hydroxytestosterone.

The highest absolute value for each parameter in Fig. 2 of the main text was normalized to 100 % (mutant III shows the highest value for all parameters). Using the data obtained from the additivity equations (Supplementary Tables 3-9), we then calculated the theoretical values for each binary combination (indicated in empty bars with stripes) and compared it to the experimental data for each mutant (solid bars) in each pathway. See Supplementary Figure 14.

From the 6 possible pathways, four are non-accessible (67 %) because no improvements in fitness are observed. Fitness is defined according to the 7 different parameters of Supplementary Figure 14. Below, a and f exemplify an inaccessible and accessible pathway, respectively.

In the first step, the introduction of mutation R47I (--- + I--) shows additive effects for selectivity and coupling efficiency, and slightly antagonistic effects for the remaining parameters; however, the pathway is not accessible because no catalytic improvement was obtained compared to the parent enzyme (a). Conversely, introduction of mutation Y51I (--- + --I) results in synergistic effects on all parameters, which are particularly strong for selectivity and CE by >2-fold (f).

In the second step, combining mutation T49I with R47I (I-- + -I- = II-) provokes moderate cooperative effects on selectivity and coupling efficiency without affecting all the remaining parameters (a). In contrast, combining the same mutation T49I with Y51I (--I + -I- = II-) results in synergistic effects on all parameters by 2-5-fold (f).

Finally, introducing mutation Y51I into mutant II- (II- + --I = III) results in pronounced cooperative effects by ~1.5 to >7-fold on all parameters (a), whereas addition of mutation R47I to -II (-II + I-- = III) causes less pronounced synergistic effects in all parameters, with conversion, CE and TTN showing almost no improvements (f).

This brief analysis shows that although all mutations are necessary for the emergence of positive epistatic effects, some pathways are more accessible than others depending on the fitness trait. For example, pathways starting with mutation Y51I show a high degree of selectivity (e, f), which is not observed in the other 4 pathways. Likewise, combining the previous mutation with T49I in two different pathways results in significant improvements in NCR, PFR and TTF (d, f).

Supplementary Note 8: Conformational population analysis.

The conformational population analysis displayed in Figure 6c, 7a and Supplementary Fig 13 was obtained by applying the dimensionality reduction technique Principal Component Analysis (PCA) on the whole dataset of the apo trajectories (three replicas of 600 ns cMD for each mutant, i.e. an accumulated simulation time of 14.4 μs) considering the distances between all alpha carbons using the PyEMMA[35] software. The conformational population analysis obtained from PC1, PC2, and PC3 was able to successfully discriminate between 2- and 15-selective mutants. To further evaluate the physical meaning of the conformational changes involved in the first Principal Component (PC1) in Figure 6b,c, the PC1/PC3 space was used as it provided a better separation of 2- and 15-selective variants. The following procedure was followed: (i) the trajectory of the two mutant -I- (replica2) and III (replica3) were chosen as they exhibit the major variation in conformation along PC1, being located at its minimum and maximum values, respectively; (ii) the PCA dataset of each of the two mutant were clusterized into 200 clusters; (iii) the cluster located closer to the minimum of the conformational populational analysis was chosen as a representative structure for the overlay.

Supplementary Note 9: Shortest Path Map analysis.

The initial step of the Shortest Path Map (SPM) analysis generates a map based on the mean distances and correlation values computed along the MD simulation. For any residue of the protein a node is created and centered on its C_α if a mean distance shorter than 6 Å is explored with other residues along the simulation time. Subsequently, the length of the line connecting the nodes is drawn according to the correlation value of the corresponding residues (d_{ij}=-log $|C_{ij}|$). Larger correlation values (closer to |1|) will have shorter edge distances, while less correlated residue pairs (values closer to 0) will have longer edges distances. The Dijkstra algorithm is finally applied to identify the shortest path lengths. The algorithm goes through all the nodes of the graph and determines which is the shortest path to go from the first until the last protein residue. Hence, the method identifies the shorter edges of the graph (i.e. highly correlated), that are more frequently used for going through all residues of the protein (i.e. they are more central for the communication pathway). The SPM method is discussed in details in our recent work.[36]

Supplementary Note 10: Additional Packages.

The Kernel Density Estimation (KDE) plots in Fig. 3 and Supplementary Figures 6 and 7 were prepared monitoring the geometrical parameters (i.e. (haem)O···2/15C(TES) distance and (haem)O···H–2/15C(TES) angle) from cMD trajectories with **1** bound in pose 2 and pose 15 in the active site of the different mutants, using the Seaborn python library.[37] The principal component 2 (pc2) shown in Figures 4d, 6b and Supplementary Figure 12 was computed from the cumulated apo trajectory of the different mutants using the Principal Component Analysis (PCA) available in the Bio3D package.[38,39] The volumes of (i) the active site in mutants --- and III (Supplementary Figure 8) and (ii) the area of the access channel 2 in mutant -I-, III (Figure 7c) were calculated with the POVME2 software,[40] with a 1.0 Å grid spacing and 1.35 Å distance cutoff. In (i) the calculation was done on the most populated clusters of the joint apo trajectories of the two mutants, with a sphere of 6 Å as the including region, whereas in (ii) the structures used were those arising from the conformational population analysis (Supplementary Note 8, *vide supra*) with a sphere of 4 Å as the including region. All the images of the P450 mutants shown were done using the PyMOL software.[41]

Supplementary Figures

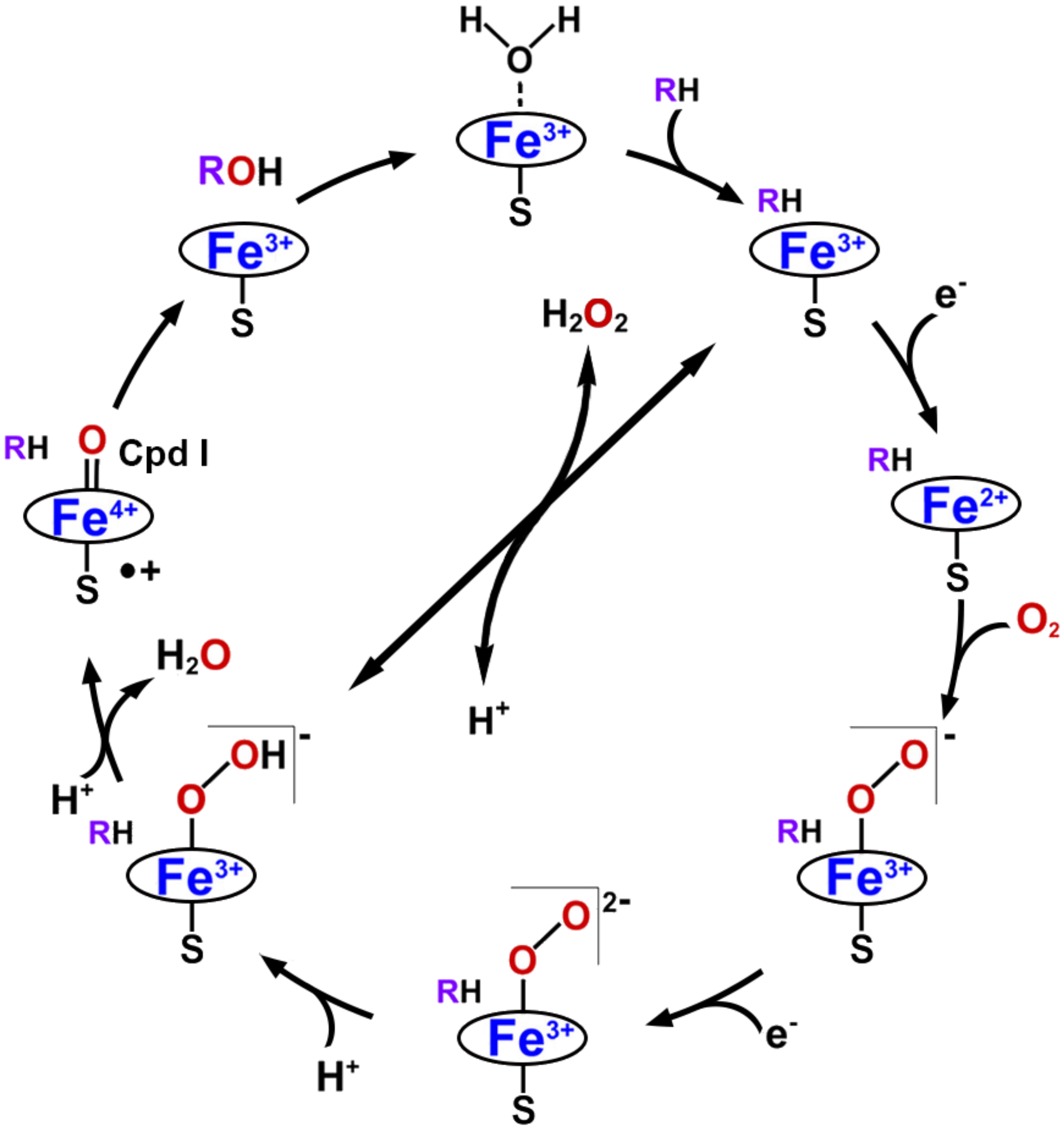

Supplementary Figure 1: Catalytic mechanism for aliphatic C-H hydroxylation catalyzed by P450s.

Graphical representation of the intermediate stages leading to substrate (RH) hydroxylation in the catalytic cycle of cytochrome P450. Redrawn from Ref[42].

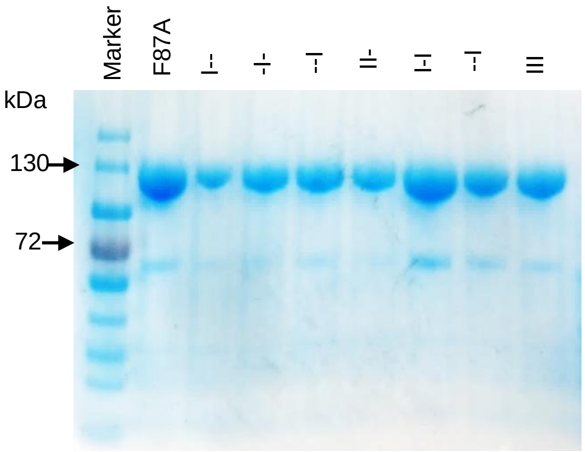

Supplementary Figure 2: SDS-PAGE analysis of purified mutants.

All N-terminal his-tagged proteins show purity above 80-90%. The fractions with purified protein from a single culture were pooled and concentrated for each mutant. One purification was done per protein variant. All samples were processed in parallel and loaded onto the same protein gel.

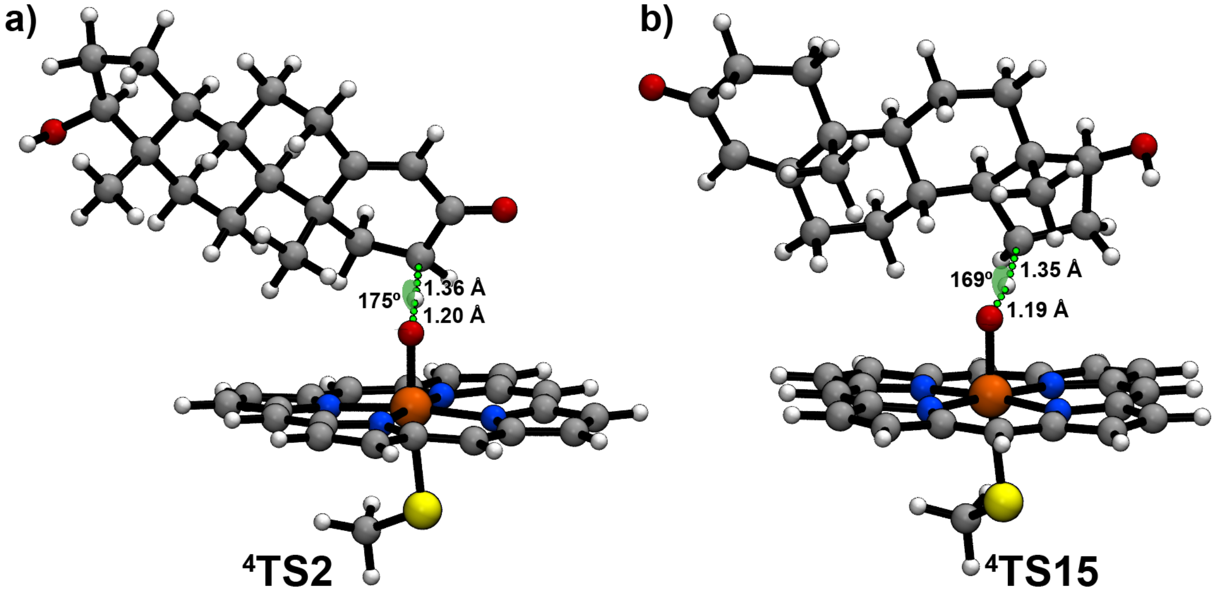

Supplementary Figure 3: QM transition-states for the C–H abstraction in 1 from (a) C-2 (b) C-15.

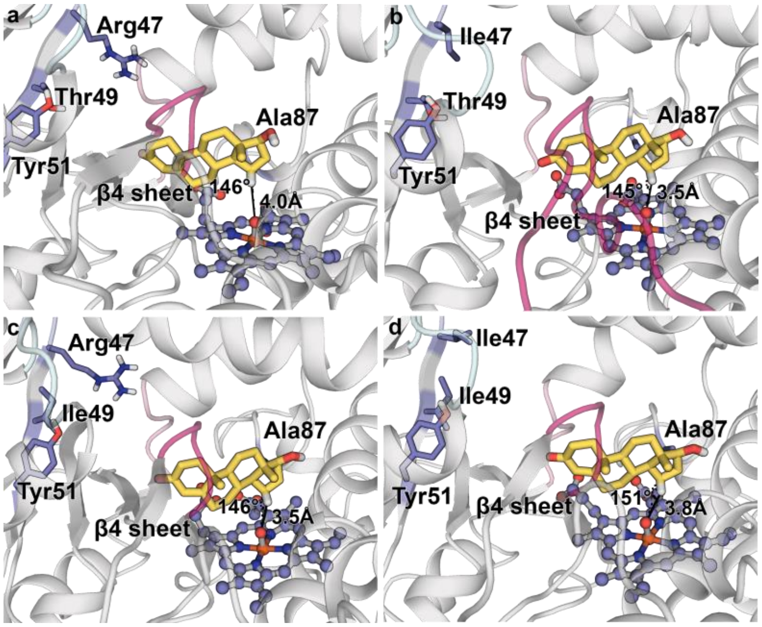

Supplementary Figure 4: Snapshot of 1 binding pose of during MD simulations in 15β-selective mutants.

Mutants (a) ---; (b) I--; (c) -I- and (d) II- with distances measured between the oxygen atom of the Fe=O and the C15 atom of **1** and angles formed by O(Fe=O) – (**1**)-H(C15) – (**1**)-C(15).

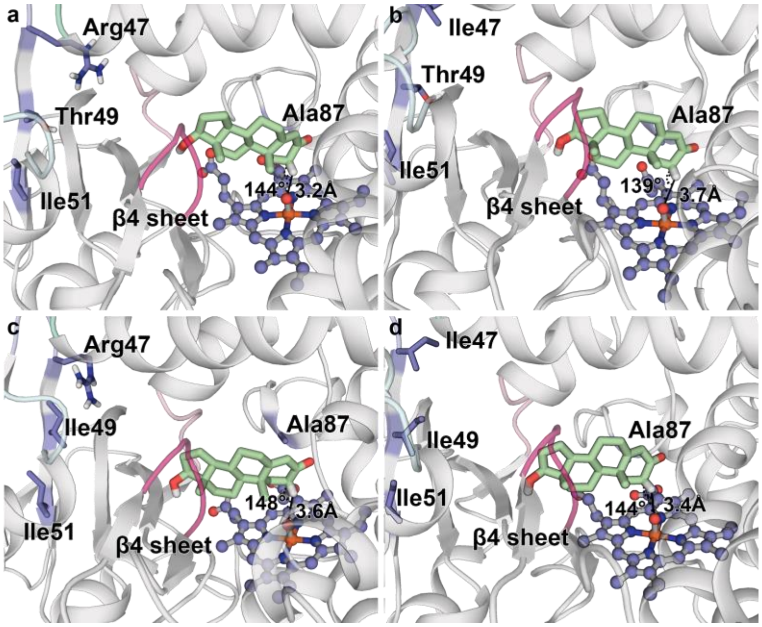

Supplementary Figure 5: Snapshot of binding pose during MD simulations in 2β-selective mutants.

Mutants (a) I--; (b) I-I; (c) -II and (d) III with distances measured between the oxygen atom of the Fe=O and the C2 atom of **1** and angles formed by O(Fe=O) – (**1**)-H(C2) – (**1**)-C(2).

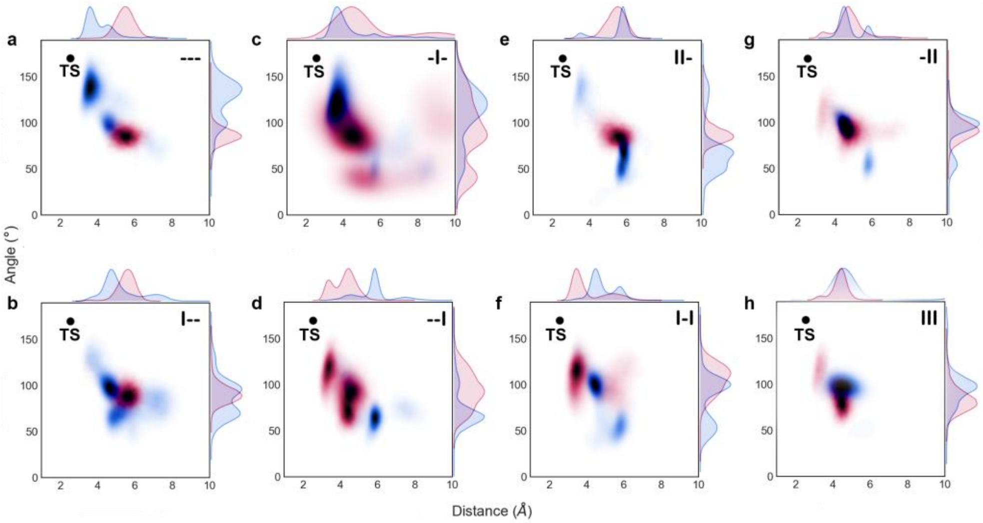

Supplementary Figure 6: KDE plot of the second replica dataset.

Distances determined between the oxygen atom of the Fe=O and the carbon atom (C2 or C15) of **1** (x-axis) and angles formed by O(Fe=O) – (**1**)-H(C2/15) – (**1**)-C(2/15) (y axis) from MD trajectories of mutants (a) ---, (b) I--, (c) -I-, (d) --I, (e) II-, (f) I-I, (g) -II and (h) III. Geometric parameters measured for carbon 2 (C2) and carbon 15 (C15) are shown in red and blue, respectively. The ideal distance and angle for the TS (black dot) corresponds to the Density Functional Theory (DFT) optimized geometry for the C–H abstraction by Fe=O using a truncated computational model.

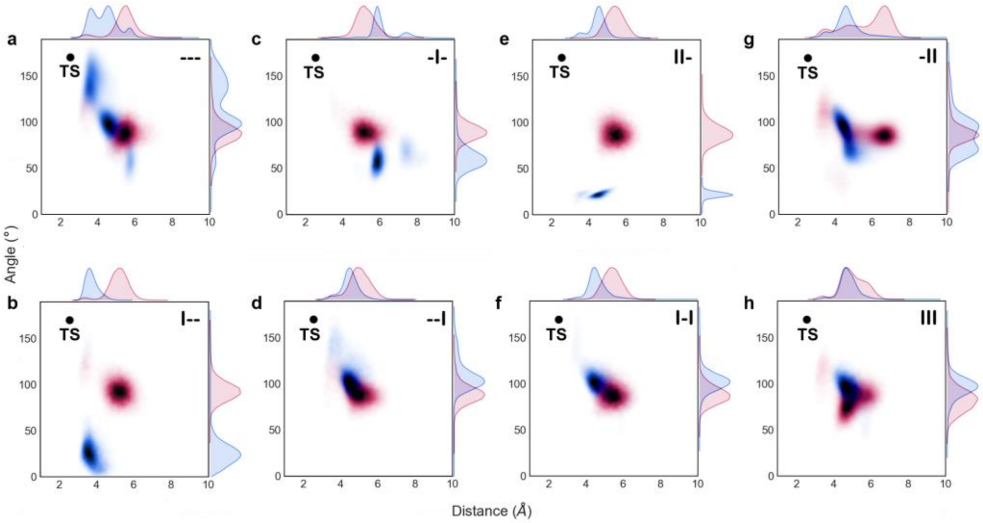

Supplementary Figure 7: KDE plot of the third replica dataset.

Distances determined between the oxygen atom of the Fe=O and the carbon atom (C2 or C15) of **1** (x-axis) and angles formed by O(Fe=O) – (**1**)-H(C2/15) – (**1**)-C(2/15) (y axis) from MD trajectories of mutants (a) ---, (b) I--, (c) -I-, (d) --I, (e) II-, (f) I-I, (g) -II and (h) III. Geometric parameters measured for carbon 2 (C2) and carbon 15 (C15) are shown in red and blue, respectively. The ideal distance and angle for the TS (black dot) corresponds to the Density Functional Theory (DFT) optimized geometry for the C–H abstraction by Fe=O using a truncated computational model.

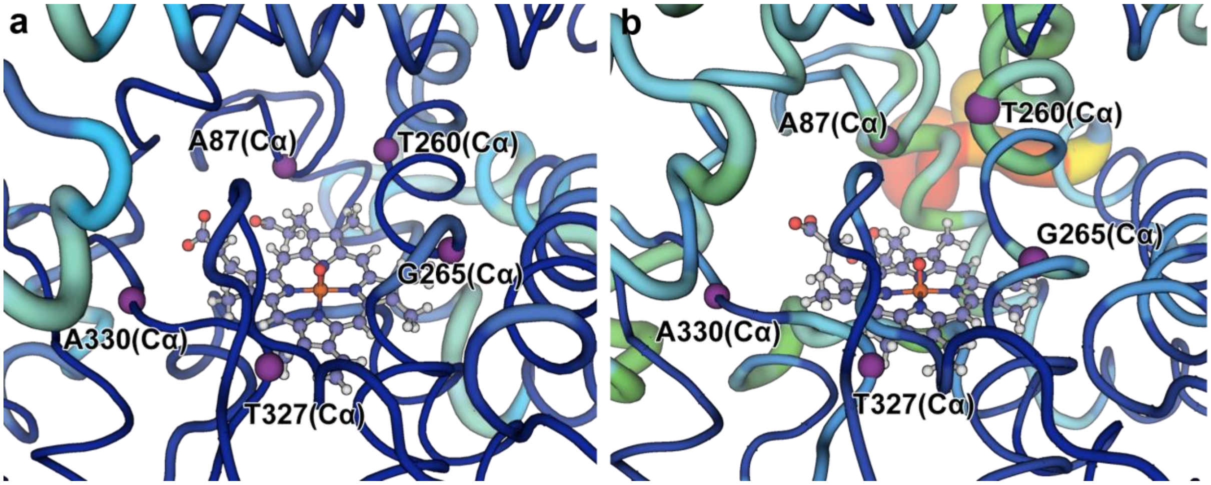

Supplementary Figure 8: PC Analysis (pc2) of the substrate-bound mutant (a) -II and (b) III.

The thickness of the line is proportional to the motion and the colour scale varies from blue (minimum motion) to red (maximum motion). C_α of selected residues are shown in purple spheres.

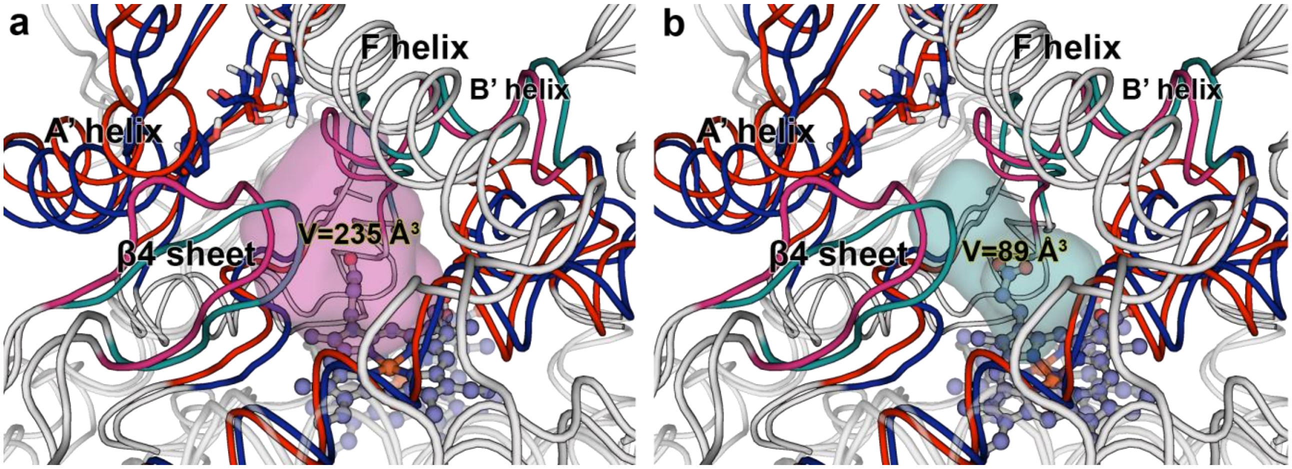

Supplementary Figure 9: Computed active site volume for mutants (a) III and (b) ---.

Mutant I-- is shown in blue, whereas the evolved mutant III is coloured in red. The F-G loop and the β1 sheet / active site volume are highlighted in teal and magenta colour for -I- and III, respectively. Active site volume calculations: The volumes of the active site in mutants --- and III were calculated with the POVME2 software,[40] with a 1.0 Å grid spacing and 1.35 Å distance cutoff. The calculation was done on the most populated clusters of the joint apo trajectories of the two mutants, with a sphere of 6 Å as the including region.

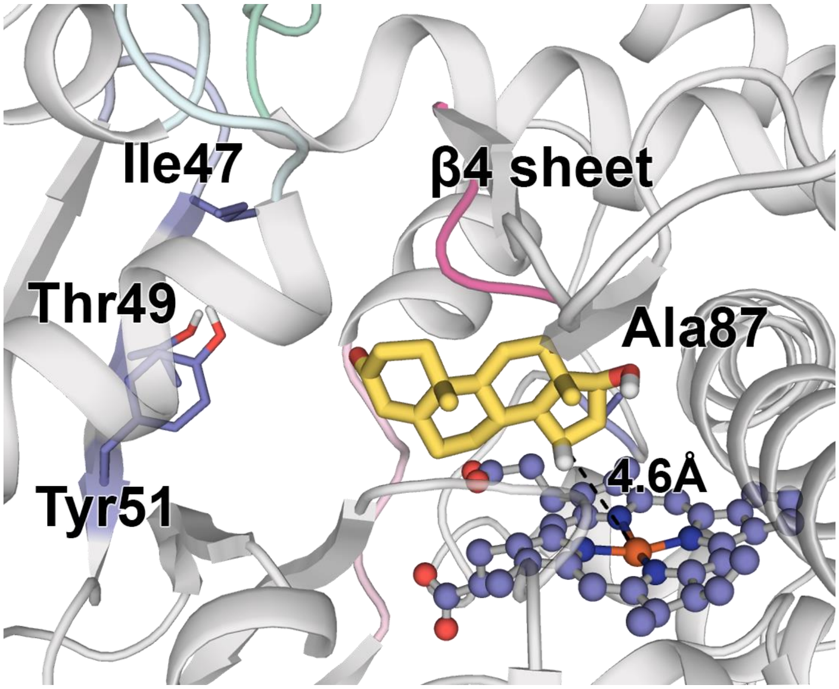

Supplementary Figure 10: Final pose of 1 in the aMD binding trajectory of I--.

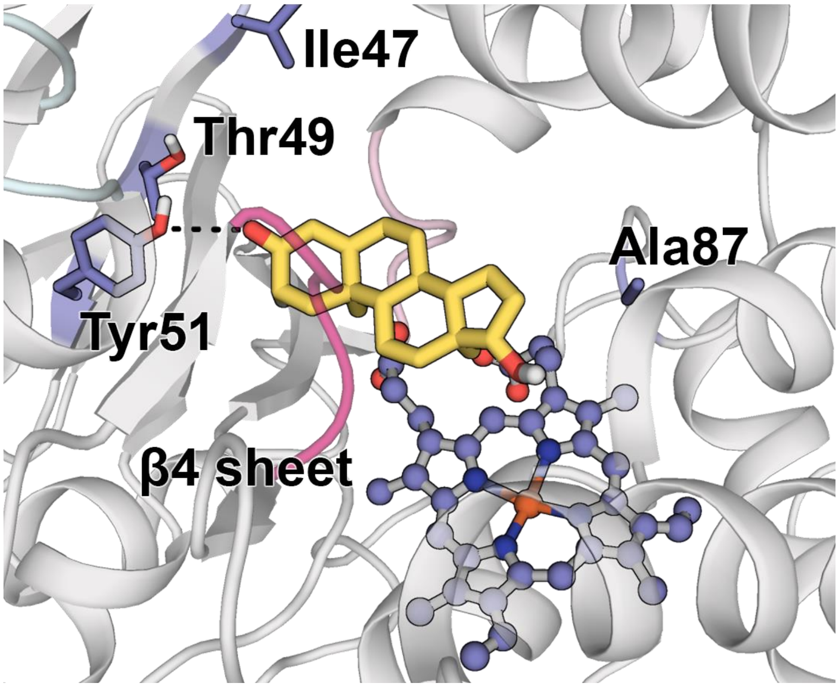

Supplementary Figure 11: (1)CO-Y51 polar interaction interaction.

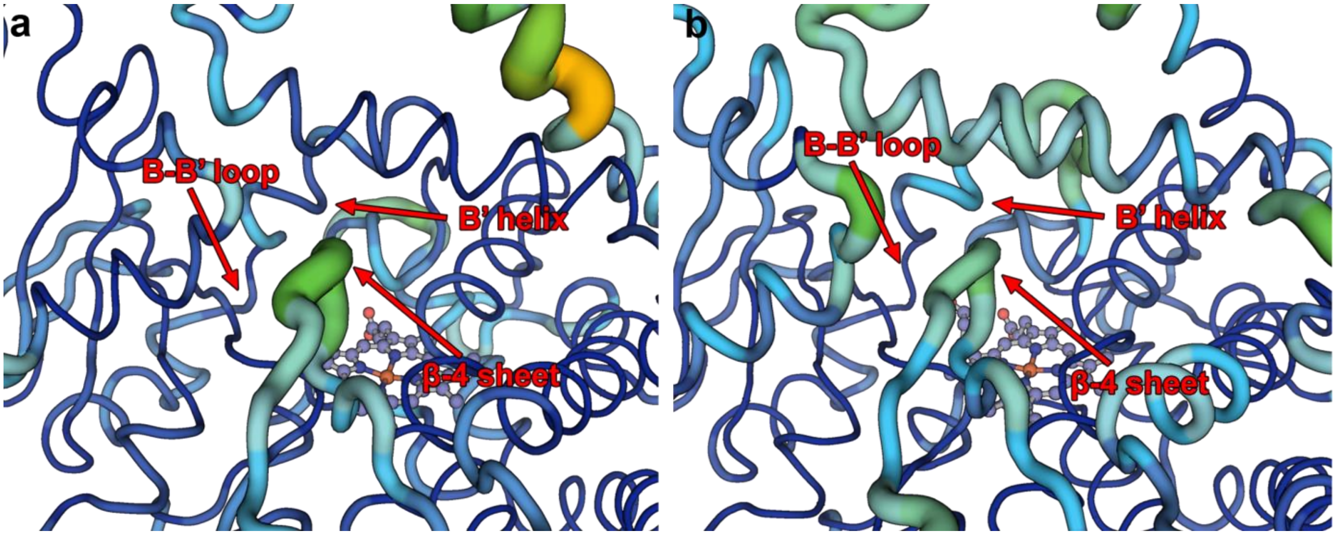

Supplementary Figure 12: PC Analysis (pc2) of substrate free mutant (a) III and (b) -II.

The thickness of the line is proportional to the motion and the colour scale varies from blue (minimum motion) to red (maximum motion). C_α of selected residues are shown in purple spheres.

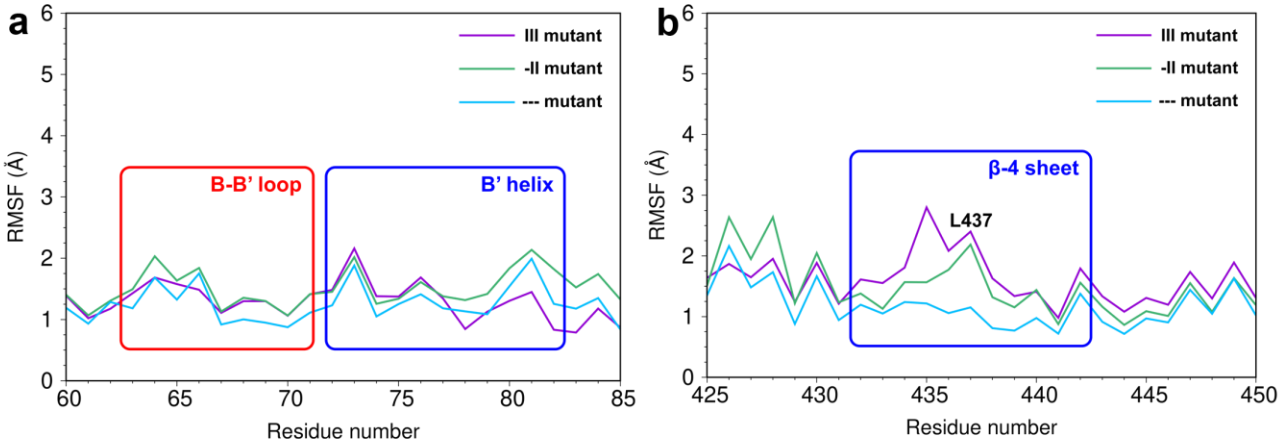

Supplementary Figure 13: RMSF of selected back-bone elements in mutants ---, -II and III.

Root-mean square fluctuation (RMSF) measured from the simulations in the absence of substrate for variant --- (in cyan), -II (green), and III (purple) for a) B-B' loop region (highlighted with a red box) and B'-helix (blue box), and b) β4 sheet (blue box). Variants III and -II show higher RMSF values of the β4 sheet region compared to ---, whereas the RMSF values are rather comparable for all enzymes over the B-B' loop and B' helix regions.

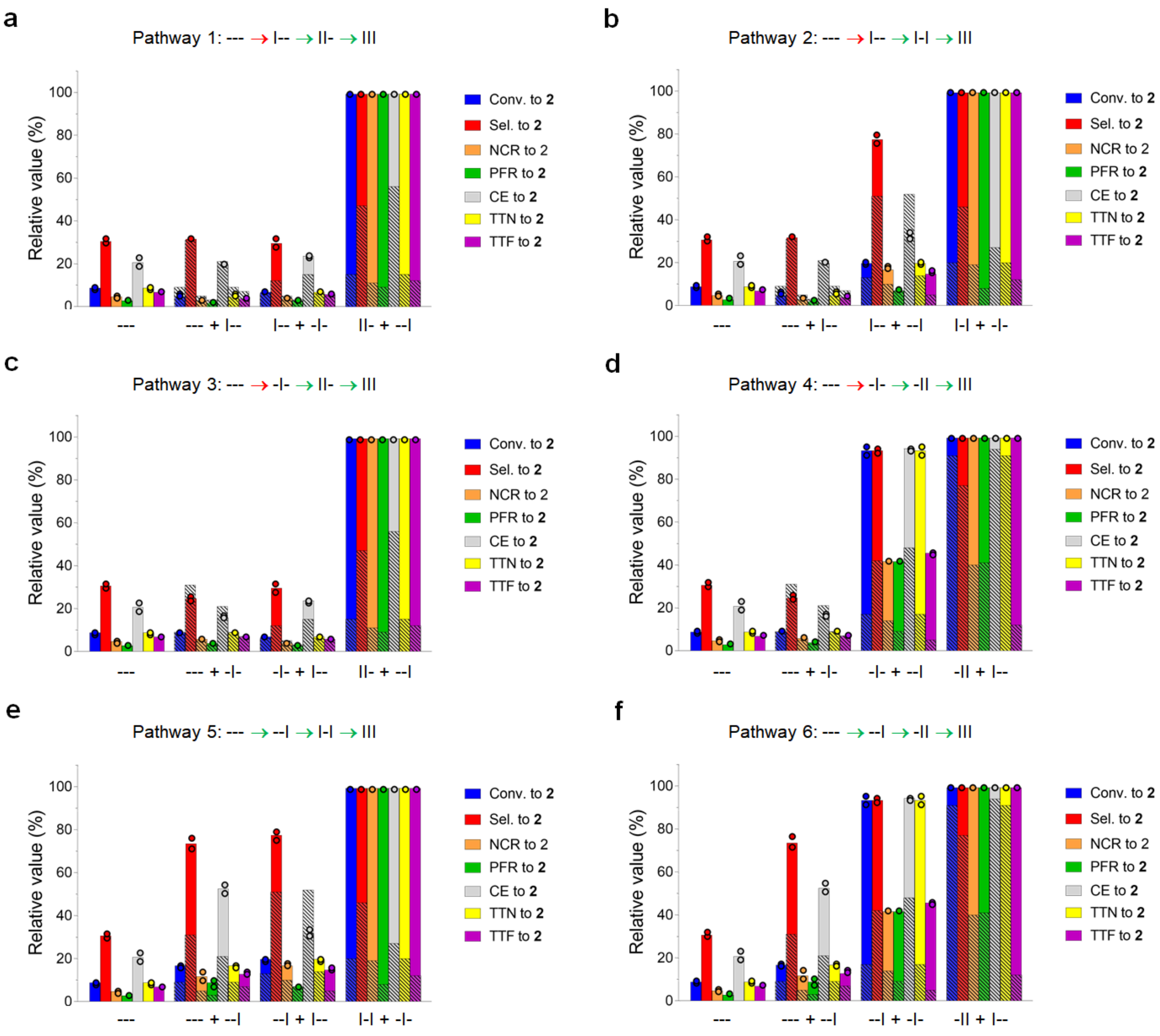

Supplementary Figure 14: Pathway accessibility for multiple enzymatic parameters towards 2β-hydroxytestosterone.

Stepwise multiparametric pathway analysis. Pathway 1 (a) or 2 (b) start with mutant F87A towards mutation R47I generating double mutant R47I/F87A. This step has no improvement and/or decreases the value of all the parameters compared to the parent enzyme F87A (red arrow) but in the second and third step, the individual introduction of mutation T49I or Y51I and Y51I or T49I, respectively, improves all parameters (green arrow). Pathway 3 (c) or 4 (d) begin with mutation T49I, while pathway 5 (e) or 6 (f) start with mutation Y51I. Green arrows indicate the absence of local minima. The experimental data for each mutant is shown in solid bars and normalized to 100 % according to the highest value. The expected theoretical values (using additivity equations) are indicated in empty bars with stripes. The data represent the average of two independent experiments (n = 2). Source data are provided with this paper.

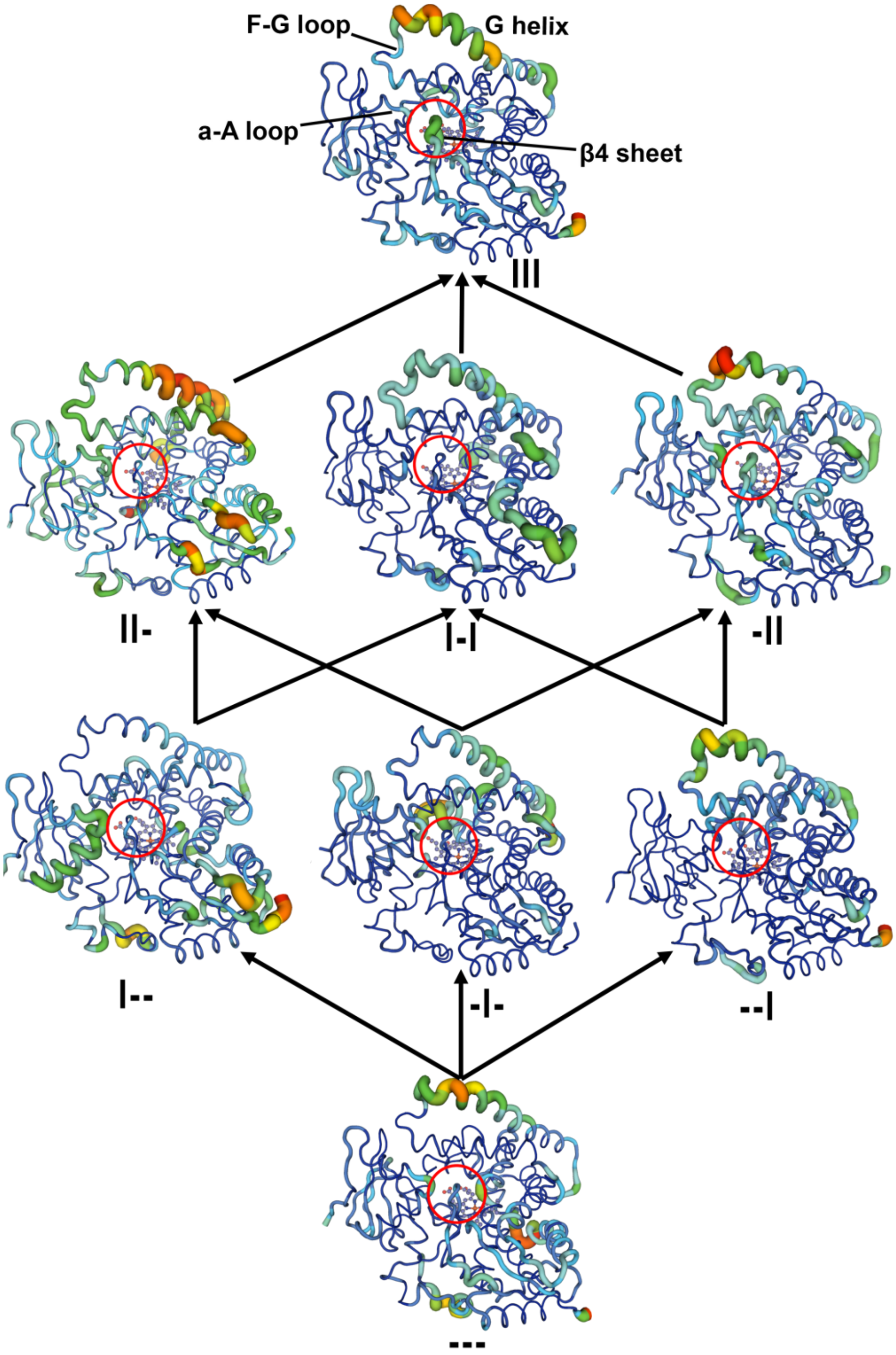

Supplementary Figure 15: PC Analysis (pc2) of apo-state mutated enzymes along the fitness landscape pathways.

The thickness of the line is proportional to the motion and the colour scale varies from blue (minimum motion) to red (maximum motion).

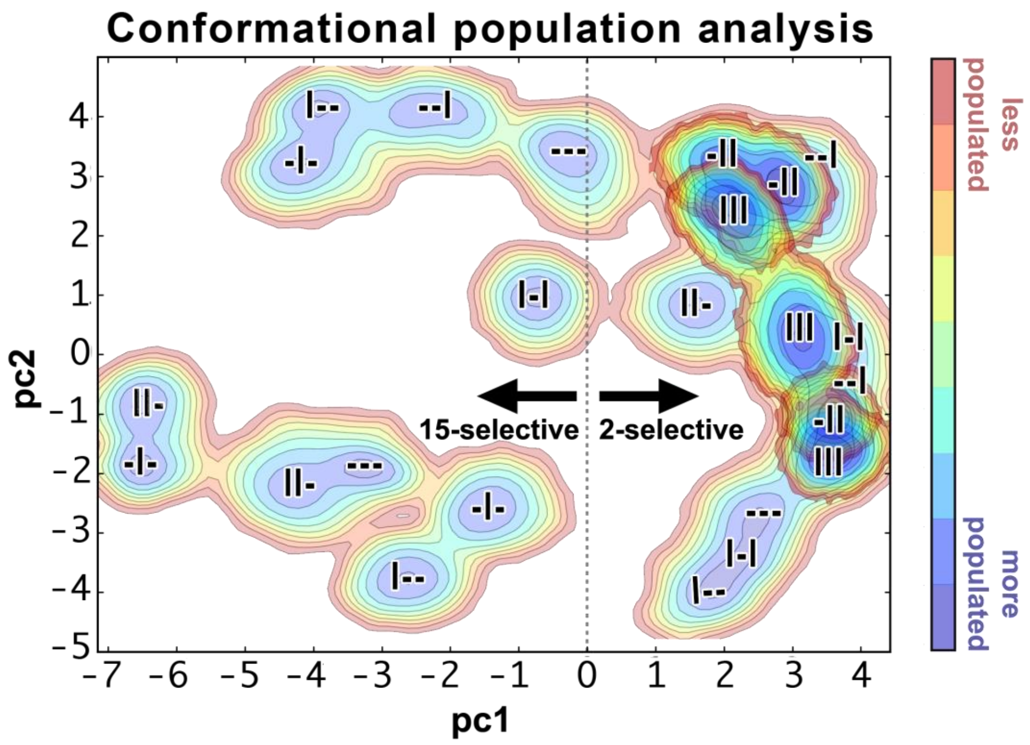

Supplementary Figure 16: Conformational population analysis (pc1, pc2) of deconvoluted mutants.

The free energies of 2β-selective mutants -II and III are highlighted.

Supplementary Tables

Supplementary Table 1: Kinetic profiles of deconvoluted mutants.

Entry	Mutant[a]	Conc.[b] (µM)	1 Conv. (%)[c]	2-Selectivity[d]		Time[e] (min)	NCR[f] (µmol / µmol x min)	NLR[g] (µmol / µmol x min)	CE[h] (%)	PFR[i] (µmol / µmol x min)	TTN[j] (µmol/ µmol)	TTF[k] (µmol / µmol x min)
				HPLC (%)	ΔΔG‡ (kJ x mol)							
1	---	1.5	10 ± 0	28 ± 1	-2.323 ± 0.124	5.0 ± 0	52 ± 1	104	25 ± 2	13 ± 0	194 ± 11	39 ± 2
2	I--	1.3	6 ± 0	29 ± 0	-2.229 ± 0.051	5.0 ± 0	32 ± 1	71	23 ± 0	7 ± 0	120 ± 1	24 ± 0
3	-I-	0.9	12 ± 0	23 ± 1	-3.015 ± 0.136	5.0 ± 0	89 ± 3	117	24 ± 1	21 ± 1	246 ± 6	49 ± 1
4	--I	1.9	8 ± 0	67 ± 2	1.802 ± 0.222	5.0 ± 0	57 ± 8	63	26 ± 1	15 ± 2	157 ± 8	31 ± 2
5	II-	1.5	8 ± 0	27 ± 2	-2.494 ± 0.259	5.2 ± 0.1	50 ± 1	60	30 ± 0	15 ± 0	168 ± 3	33 ± 0
6	I-I	1.3	9 ± 0	71 ± 1	2.189 ± 0.119	5.1 ± 0.1	78 ± 2	149	15 ± 0	12 ± 0	175 ± 6	34 ± 1
7	-II	3.2	35 ± 1	85 ± 1	4.358 ± 0.192	8.3 ± 0.1	157 ± 3	70	37 ± 1	58 ± 1	700 ± 16	85 ± 1
8	III	2.0	35 ± 0	91 ± 0	5.594 ± 0.052	4.0 ± 0	354 ± 7	117	37 ± 1	131 ± 3	700 ± 1	174 ± 0

The enzymatic reactions were performed using 100 nM enzyme, 0.2 mM testosterone (**1**) as substrate and 1 % DMF as cosolvent and 0.24 mM NADPH in potassium phosphate (KPi) buffer (pH 8.0) at 25 °C until NADPH depletion, which was measured at 340 nm. Data represent the average ± SEM of two independent experiments (*n* = 2).
a) Mutant: (---) = F87A, (-) = absence of mutation(s) at position(s) R47, T49 and/or Y51, III = R47I/T49I/Y51I/F87A.
b) Concentration of purified enzymes after purification was determined by CO difference spectrum analysis.
c) Conversion of testosterone to all products (total conversion) after NADPH depletion as measured by HPLC (%)
d) ΔΔG‡ (KJ/mol) = Ln (2β-hydroxytestosterone / all other regio- and -stereoisomers) x (298 K x 0.001 x 8.31441 J mol^{-1} K^{-1}).
e) Time after NADPH depletion.
f) NADPH consumption rate (NCR) is the total µmol NADPH consumed (to convert substrate into products) per µmol enzyme per min.
g) NADPH leak rate (NLR) is µmol NADPH consumed per µmol enzyme per min in the absence of substrate (*n*=1).
h) Coupling efficiency (CE) is the ratio between total consumption of substrate vs. NADPH.
i) Product formation rate (PFR) is determined by the multiplication of NCR with CE and has same units as NCR.
j) Total turnover number (TTN) refers to total amount of products formed and divided by the enzyme concentration after NADPH depletion.
k) Total turnover frequency (TTF) is calculated by dividing the TTN values above by total time (after NADPH was depleted).

Supplementary Table 2: Energies, thermal corrections and free energies of the QM structures calculated for H-abstraction catalyzed by haem.

Structure[a]	E_{el}[b]	E_{el}[c]	G correction[d]	G[e]	ΔG
	(Hartree)[f]	(Hartree)[f]	(Hartree/particle)[f]	(Hartree)[f]	(kcal/mol)
**^{4}cpdI**	-1625.181930	-1625.492613	0.267701	-1625.224912	
**1**	-891.402149	-891.636974	0.397895	-891.239079	
**^{4}RC[g]**	- 2516.584079	-2517.129587	0.665596	-2516.463991	
**^{4}TS2**	-2516.560005	-2517.123777	0.681106	-2516.442671	13.4
**^{4}TS15**	-2516.559394	-2517.123139	0.680743	-2516.442396	13.6

a) All the calculation were performed in the quartet state under the unrestricted formalism exept for 1 (singlet, restricted).
b) Enegy obtained from geometries optimized at the B3LYP-D3 / 6-31G* + LANL2DZ(Fe) level of theory.
c) Energy obtained from single point calculation at the B3LYP-D3 / 6-311G** + LANL2DZ(Fe) level of theory on top of the optimized geometry.
d) Thermal correction at 298.15 K obtained from frequency calculation on top the optimized geometry at the same level of theory.
e) Energy calculated as Eelc + G correction.
f) 1 Hartree = 627.5 kcal/mol.
g) Energy and thermal correction calculated as 4cpdI + 1.

Supplementary Table 3: Additivity calculations for substrate conversion to 2β-hydroxytestosterone.

Combination	R47I	T49I	Y51I	Mutations	Exp. Mean	Exp. SEM	Theor. Mean	Theor. SEM	Exp. - Theor. (Mean)	Result
1	+	+	-	(2, 3)	2.3	0.0	1.8	0.0	0.4	+ Sign epistasis
2	-	+	+	(3, 4)	29.9	0.5	5.4	0.2	24.5	+ Magnitude epistasis
3	+	-	+	(2, 4)	6.2	0.2	4.3	0.2	1.9	+ Sign epistasis
4	+	+	+	(2, 7)	31.8	0.0	28.9	0.5	2.9	+ Sign epistasis
5	+	+	+	(4, 5)	31.8	0.0	4.8	0.2	26.9	+ Sign epistasis
6	+	+	+	(3, 6)	31.8	0.0	6.3	0.2	25.5	+ Magnitude epistasis
7	+	+	+	(2, 3, 4)	31.8	0.0	4.4	0.2	27.4	+ Sign epistasis

The signs +/- mean that the respective mutation is present/absent. Mutation 2: R47I; Mutation 3: T49I; Mutation 4: Y51I. Mutation 5: T49I/Y51I; Mutation 6: R47I/Y51I; Mutation 7: R47I/T49I. The values are calculated with original data from **Supplementary Table 1** and represent the average of two independent experiments (n=2).

Supplementary Table 4: Additivity calculations for 2β-hydroxytestosterone selectivity.

Combination	R47I	T49I	Y51I	Mutations	Exp. Mean	Exp. SEM	Theor. Mean	Theor. SEM	Exp. - Theor. (Mean)	Result
1	+	+	-	(2, 3)	-2.5	0.2	-5.2	0.1	2.8	+ Magnitude epistasis
2	-	+	+	(3, 4)	4.4	0.1	-1.2	0.2	5.6	+ Sign epistasis
3	+	-	+	(2, 4)	2.2	0.1	-0.4	0.2	2.6	+ Sign epistasis
4	+	+	+	(2, 7)	5.6	0.0	2.1	0.1	3.5	+ Sign epistasis
5	+	+	+	(4, 5)	5.6	0.0	-0.7	0.2	6.3	+ Sign epistasis
6	+	+	+	(3, 6)	5.6	0.0	-0.8	0.2	6.4	+ Sign epistasis
7	+	+	+	(2, 3, 4)	5.6	0.0	-3.5	0.2	9.0	+ Sign epistasis

The signs +/- mean that the respective mutation is present/absent. Mutation 2: R47I; Mutation 3: T49I; Mutation 4: Y51I. Mutation 5: T49I/Y51I; Mutation 6: R47I/Y51I; Mutation 7: R47I/T49I. The values are calculated with original data from **Supplementary Table 1** and represent the average of two independent experiments (n=2).

Supplementary Table 5: Additivity calculations for NADPH consumption rate (NCR) to 2β-hydroxytestosterone.

Combination	R47I	T49I	Y51I	Mutations	Exp. Mean	Exp. SEM	Theor. Mean	Theor. SEM	Exp. - Theor. (Mean)	Result
1	+	+	-	(2, 3)	13.5	0.2	14.8	0.5	-1.3	- Sign epistasis
2	-	+	+	(3, 4)	134.2	1.9	43.7	3.9	90.5	+ Magnitude epistasis
3	+	-	+	(2, 4)	55.2	0.9	32.6	3.9	22.6	+ Sign epistasis
4	+	+	+	(2, 7)	320.7	4.6	128.7	1.9	192.0	+ Sign epistasis
5	+	+	+	(4, 5)	320.7	4.6	36.9	3.9	283.9	+ Sign epistasis
6	+	+	+	(3, 6)	320.7	4.6	60.8	1.0	259.9	+ Magnitude epistasis
7	+	+	+	(2, 3, 4)	320.7	4.6	38.2	3.9	282.5	+ Sign epistasis

The signs +/- mean that the respective mutation is present/absent. Mutation 2: R47I; Mutation 3: T49I; Mutation 4: Y51I. Mutation 5: T49I/Y51I; Mutation 6: R47I/Y51I; Mutation 7: R47I/T49I. The values are calculated with original data from **Supplementary Table 1** and represent the average of two independent experiments (n=2).

Supplementary Table 6: Additivity calculations for product formation rate (PFR) to 2β-hydroxytestosterone.

Combination	R47I	T49I	Y51I	Mutations	Exp. Mean	Exp. SEM	Theor. Mean	Theor. SEM	Exp. - Theor. (Mean)	Result
1	+	+	-	(2, 3)	4.0	0.1	3.3	0.1	0.7	+ Sign epistasis
2	-	+	+	(3, 4)	49.9	0.7	11.2	1.0	38.7	+ Magnitude epistasis
3	+	-	+	(2, 4)	8.4	0.1	8.5	1.0	-0.2	Additive
4	+	+	+	(2, 7)	118.7	1.7	48.4	0.7	70.3	+ Sign epistasis
5	+	+	+	(4, 5)	118.7	1.7	10.4	1.0	108.3	+ Magnitude epistasis
6	+	+	+	(3, 6)	118.7	1.7	9.5	0.2	109.1	+ Magnitude epistasis
7	+	+	+	(2, 3, 4)	118.7	1.7	9.7	1.0	109.0	+ Sign epistasis

The signs +/- mean that the respective mutation is present/absent. Mutation 2: R47I; Mutation 3: T49I; Mutation 4: Y51I. Mutation 5: T49I/Y51I; Mutation 6: R47I/Y51I; Mutation 7: R47I/T49I. The values are calculated with original data from **Supplementary Table 1** and represent the average of two independent experiments (n=2).

Supplementary Table 7: Additivity calculations for coupling efficiency (CE) towards 2β-hydroxytestosterone.

Combination	R47I	T49I	Y51I	Mutations	Exp. Mean	Exp. SEM	Theor. Mean	Theor. SEM	Exp. - Theor. (Mean)	Result
1	+	+	-	(2, 3)	7.9	0.0	5.2	0.2	2.8	+ Reciprocal sign epistasis
2	-	+	+	(3, 4)	31.7	0.5	16.2	0.3	15.5	+ Sign epistasis
3	+	-	+	(2, 4)	10.7	0.2	17.5	0.3	-6.8	- Sign epistasis
4	+	+	+	(2, 7)	33.5	0.4	31.4	0.5	2.1	+ Sign epistasis
5	+	+	+	(4, 5)	33.5	0.4	18.7	0.3	14.8	+ Magnitude epistasis
6	+	+	+	(3, 6)	33.5	0.4	9.2	0.3	24.3	+ Sign epistasis
7	+	+	+	(2, 3, 4)	33.5	0.4	15.9	0.3	17.6	+ Sign epistasis

The signs +/- mean that the respective mutation is present/absent. Mutation 2: R47I; Mutation 3: T49I; Mutation 4: Y51I. Mutation 5: T49I/Y51I; Mutation 6: R47I/Y51I; Mutation 7: R47I/T49I. The values are calculated with original data from **Supplementary Table 1** and represent the average of two independent experiments (n=2).

Supplementary Table 8: Additivity calculations for TTN towards 2β-hydroxytestosterone.

Combination	R47I	T49I	Y51I	Mutations	Exp. Mean	Exp. SEM	Theor. Mean	Theor. SEM	Exp. - Theor. (Mean)	Result
1	+	+	-	(2, 3)	45.0	1.6	36.2	2.4	8.8	+ Sign epistasis
2	-	+	+	(3, 4)	597.5	14.1	107.5	6.4	490.1	+ Magnitude epistasis
3	+	-	+	(2, 4)	124.0	4.8	85.8	6.0	38.3	+ Sign epistasis
4	+	+	+	(2, 7)	635.0	0.8	577.5	14.1	57.5	+ Sign epistasis
5	+	+	+	(4, 5)	635.0	0.8	96.2	6.2	538.8	+ Sign epistasis
6	+	+	+	(3, 6)	635.0	0.8	125.7	5.4	509.3	+ Magnitude epistasis
7	+	+	+	(2, 3, 4)	635.0	0.8	87.4	6.5	547.6	+ Sign epistasis

The signs +/- mean that the respective mutation is present/absent. Mutation 2: R47I; Mutation 3: T49I; Mutation 4: Y51I. Mutation 5: T49I/Y51I; Mutation 6: R47I/Y51I; Mutation 7: R47I/T49I. The values are calculated with original data from **Supplementary Table 1** and represent the average of two independent experiments (n=2).

Supplementary Table 9: Additivity calculations for TTF towards 2β-hydroxytestosterone.

Combination	R47I	T49I	Y51I	Mutations	Exp. Mean	Exp. SEM	Theor. Mean	Theor. SEM	Exp. - Theor. (Mean)	Result
1	+	+	-	(2, 3)	8.7	0.4	7.2	0.5	1.5	+ Sign epistasis
2	-	+	+	(3, 4)	72.2	0.9	21.5	1.3	50.7	+ Magnitude epistasis
3	+	-	+	(2, 4)	24.3	0.8	17.2	1.2	7.2	+ Sign epistasis
4	+	+	+	(2, 7)	157.8	0.0	68.2	0.9	89.6	+ Sign epistasis
5	+	+	+	(4, 5)	157.8	0.0	19.0	1.3	138.8	+ Sign epistasis
6	+	+	+	(3, 6)	157.8	0.0	24.6	0.9	133.1	+ Magnitude epistasis
7	+	+	+	(2, 3, 4)	157.8	0.0	17.5	1.3	140.3	+ Sign epistasis

The signs +/- mean that the respective mutation is present/absent. Mutation 2: R47I; Mutation 3: T49I; Mutation 4: Y51I. Mutation 5: T49I/Y51I; Mutation 6: R47I/Y51I; Mutation 7: R47I/T49I. The values are calculated with original data from **Supplementary Table 1** and represent the average of two independent experiments (n=2).

Supplementary Table 10: Pathway accessibility analysis based on selectivity towards 2β-hydroxytestosterone (2).

Pathway	Mutation				ΔΔG‡ (kJ per mol)			
	0	1st	2nd	3rd	0	1st	2nd	3rd
1	---	I--	II-	III	-2.328	-2.229	-2.494	5.594
2	---	I--	I-I	III	-2.328	-2.229	2.187	5.594
3	---	-I-	II-	III	-2.328	-3.017	-2.494	5.594
4	---	-I-	-II	III	-2.328	-3.017	4.358	5.594
5	---	--I	I-I	III	-2.328	1.796	2.187	5.594
6	---	--I	-II	III	-2.328	1.796	4.358	5.594

A green or red pathway/value means that there is a respective increase or decrease of fitness in each step of the evolutionary process from parental mutant F87A (---) toward triple mutant III (R47I/T49I/Y51I/F87A). Values in orange indicate that there is neither increase nor decrease in fitness between the precedent and subsequent mutation (considering SEM), thus its pathway turns red. Conversely to red, green pathways are accessible to stepwise evolution. The values are calculated with original data from **Supplementary Table 1** and represent the average of two independent experiments (n=2).

Supplementary Table 11: Pathway accessibility analysis based on TTF towards 2β-hydroxytestosterone (2).

Pathway	Mutation				Total Turnover Number (TTN)			
	0	1st	2nd	3rd	0	1st	2nd	3rd
1	---	I--	II-	III	11	7	9	158
2	---	I--	I-I	III	11	7	24	158
3	---	-I-	II-	III	11	11	9	158
4	---	-I-	-II	III	11	11	72	158
5	---	--I	I-I	III	11	21	24	158
6	---	--I	-II	III	11	21	72	158

A green or red pathway/value means that there is a respective increase or decrease of fitness in each step of the evolutionary process from parental mutant F87A (---) toward triple mutant III (R47I/T49I/Y51I/F87A). Values in orange indicate that there is neither increase nor decrease in fitness between the precedent and subsequent mutation (considering SEM), thus its pathway turns red. Conversely to red, green pathways are accessible to stepwise evolution. The values are calculated with original data from **Supplementary Table 1** and represent the average of two independent experiments (n=2).

Supplementary Table 12: Oligonucleotides used in this study.

Code	Target	Sequence (5'-3')
I--_FW	Mutagenesis R47I	CGCCTGGT**ATC**GTAACGCGCTAC
-I-_FW	Mutagenesis T49I	GGTCGTGTA**ATC**CGCTACTTATCAAG
--I_FW	Mutagenesis Y51I	CGTGTAACGCGC**ATC**TTATCAAGTCAG
II-_FW	Mutagenesis R47I-T49I	GGT**ATC**GTA**ATC**CGCTACTTATCAAG
I-I_FW	Mutagenesis R47I-Y51I	GGT**ATC**GTAACGCGC**ATC**TTATCAAG
-II_FW	Mutagenesis T49I-Y51I	GGTCGTGTA**ATC**CGC**ATC**TTATCAAG
T7Trw_UHT	Mutagenesis with Silent MegaPrimer	GCTAGTTATTGCTCAGCGGTGGCAGCAGCC
T7	Sequencing from promoter to residue ~250	TAATACGACTCACTATAGGG
2nd-BM3	Sequencing from residue ~250 to ~500	CGAGAACATTCGCTATC
3rd-BM3	Sequencing from residue ~500 to ~750	TTAATTGTAACGGCGTC
4th-BM3	Sequencing from residue ~750 to ~1050	ATACCCGGCGTGTGAAATG

*The underlined and bold bases represent the target residue, whereas the underlined non-bold bases represent the other adjacent residues.

Supplementary References

1. Atkins, W. M. Non-Michaelis-Menten kinetics in cytochrome P450-catalyzed reactions. *Annu. Rev. Pharmacol. Toxicol.* **45**, 291–310 (2005).
2. Fox, R. J. & Clay, M. D. Catalytic effectiveness, a measure of enzyme proficiency for industrial applications. *Trends Biotechnol.* **27**, 137–40 (2009).
3. Reetz, M. T. & Sanchis, J. Constructing and analyzing the fitness landscape of an experimental evolutionary process. *ChemBioChem* **9**, 2260–2267 (2008).
4. Narayan, A. R. H. *et al.* Enzymatic hydroxylation of an unactivated methylene C-H bond guided by molecular dynamics simulations. *Nat. Chem.* **7**, 653–660 (2015).
5. Frisch, M. J. *et al.* Gaussian09 {R}evision {D}.01.
6. Lee, C., Yang, W. & Parr, R. G. Development of the Colle-Salvetti correlation-energy formula into a functional of the electron density. *Phys. Rev. B* **37**, 785–789 (1988).
7. Becke, A. D. Density-functional thermochemistry. III. The role of exact exchange. *J. Chem. Phys.* **98**, 5648–5652 (1993).
8. Hay, P. J. & Wadt, W. R. *Ab initio* effective core potentials for molecular calculations. Potentials for the transition metal atoms Sc to Hg. *J. Chem. Phys.* **82**, 270–283 (1985).
9. Cossi, M., Rega, N., Scalmani, G. & Barone, V. Energies, structures, and electronic properties of molecules in solution with the C-PCM solvation model. *J. Comput. Chem.* **24**, 669–681 (2003).
10. Grimme, S., Antony, J., Ehrlich, S. & Krieg, H. A consistent and accurate ab initio parametrization of density functional dispersion correction (DFT-D) for the 94 elements H-Pu. *J. Chem. Phys.* **132**, 154104 (2010).
11. Case, D. A. *et al.* AMBER 16. (2016).
12. Wang, J., Wolf, R. M., Caldwell, J. W., Kollman, P. A. & Case, D. A. Development and testing of a general Amber force field. *J. Comput. Chem.* **25**, 1157–1174 (2004).
13. Bayly, C. I., Cieplak, P., Cornell, W. D. & Kollman, P. A. A well-behaved electrostatic potential based method using charge restraints for deriving atomic charges: The RESP model. *J. Phys. Chem.* **97**, 10269–10280 (1993).
14. Besler, B. H., Merz, K. M. & Kollman, P. A. Atomic charges derived from semiempirical methods. *J. Comput. Chem.* **11**, 431–439 (1990).
15. Singh, U. C. & Kollman, P. A. An approach to computing electrostatic charges for molecules. *J. Comput. Chem.* **5**, 129–145 (1984).
16. Shahrokh, K., Orendt, A., Yost, G. S. & Cheatham, T. E. Quantum mechanically derived AMBER-compatible heme parameters for various states of the cytochrome P450 catalytic cycle. *J. Comput. Chem.* **33**, 119–133 (2012).
17. Anandakrishnan, R., Aguilar, B. & Onufriev, A. V. H++ 3.0: automating pK prediction and the preparation of biomolecular structures for atomistic molecular modeling and simulations. *Nucleic Acids Res.* **40**, W537-41 (2012).
18. Jorgensen, W. L., Chandrasekhar, J., Madura, J. D., Impey, R. W. & Klein, M. L. Comparison of simple potential functions for simulating liquid water. *J. Chem. Phys.* **79**, 926–935 (1983).
19. Lindorff-Larsen, K. *et al.* Improved side-chain torsion potentials for the Amber ff99SB protein force field. *Proteins Struct. Funct. Bioinforma.* **78**, 1950–1958 (2010).
20. Li, H. & Poulos, T. L. The structure of the cytochrome p450BM-3 haem domain complexed with the fatty acid substrate, palmitoleic acid. *Nat. Struct. Biol.* **4**, 140–146 (1997).
21. Davis, I. W., Arendall, W. B., Richardson, D. C. & Richardson, J. S. The backrub motion: How protein backbone shrugs when a sidechain dances. *Structure* **14**, 265–274 (2006).
22. Humphris, E. L. & Kortemme, T. Prediction of Protein-Protein Interface Sequence

Diversity Using Flexible Backbone Computational Protein Design. *Structure* **16**, 1777–1788 (2008).
23. Smith, C. A. & Kortemme, T. Backrub-Like Backbone Simulation Recapitulates Natural Protein Conformational Variability and Improves Mutant Side-Chain Prediction. *J. Mol. Biol.* **380**, 742–756 (2008).
24. Darden, T., York, D. & Pedersen, L. Particle mesh Ewald: An N·log(N) method for Ewald sums in large systems. *J. Chem. Phys.* **98**, 10089–10092 (1993).
25. Hamelberg, D., Mongan, J. & McCammon, J. A. Accelerated molecular dynamics: A promising and efficient simulation method for biomolecules. *J. Chem. Phys.* **120**, 11919–11929 (2004).
26. Hamelberg, D., De Oliveira, C. A. F. & McCammon, J. A. Sampling of slow diffusive conformational transitions with accelerated molecular dynamics. *J. Chem. Phys.* **127**, 155102 (2007).
27. Miao, Y., Feixas, F., Eun, C. & McCammon, J. A. Accelerated molecular dynamics simulations of protein folding. *J. Comput. Chem.* **36**, 1536–1549 (2015).
28. Kaltenbach, M. & Tokuriki, N. Dynamics and constraints of enzyme evolution. *J. Exp. Zool. Part B Mol. Dev. Evol.* **322**, 468–487 (2014).
29. Weinreich, D. M., Delaney, N. F., DePristo, M. A. & Hartl, D. L. Darwinian evolution can follow only very few mutational paths to fitter proteins. *Science.* **312**, 111–114 (2006).
30. Meini, M.-R., Tomatis, P. E., Weinreich, D. M. & Vila, A. J. Quantitative Description of a Protein Fitness Landscape Based on Molecular Features. *Mol. Biol. Evol.* **32**, 1774–1787 (2015).
31. Rodrigues, J. V. *et al.* Biophysical principles predict fitness landscapes of drug resistance. *Proc. Natl. Acad. Sci.* **113**, E1470–E1478 (2016).
32. Yu, H. & Dalby, P. A. Coupled molecular dynamics mediate long- and short-range epistasis between mutations that affect stability and aggregation kinetics. *Proc. Natl. Acad. Sci. U. S. A.* **115**, E11043–E11052 (2018).
33. Zhang, Z.-G., Lonsdale, R., Sanchis, J. & Reetz, M. T. Extreme Synergistic Mutational Effects in the Directed Evolution of a Baeyer–Villiger Monooxygenase as Catalyst for Asymmetric Sulfoxidation. *J. Am. Chem. Soc.* **136**, 17262–17272 (2014).
34. Calzadiaz-Ramirez, L. *et al.* In Vivo Selection for Formate Dehydrogenases with High Efficiency and Specificity toward NADP+. *ACS Catal.* **10**, 7512–7525 (2020).
35. Scherer, M. K. *et al.* PyEMMA 2: A Software Package for Estimation, Validation, and Analysis of Markov Models. *J. Chem. Theory Comput.* **11**, 5525–5542 (2015).
36. Romero-Rivera, A., Garcia-Borràs, M. & Osuna, S. Role of Conformational Dynamics in the Evolution of Retro-Aldolase Activity. *ACS Catal.* **7**, 8524–8532 (2017).
37. M., W. *et al.* Seaborn v0.9.0. (2018). doi:10.5281/zenodo.1313201
38. Grant, B. J., Rodrigues, A. P. C., ElSawy, K. M., McCammon, J. A. & Caves, L. S. D. Bio3d: an R package for the comparative analysis of protein structures. *Bioinformatics* **22**, 2695–6 (2006).
39. Skjaerven, L., Yao, X. Q., Scarabelli, G. & Grant, B. J. Integrating protein structural dynamics and evolutionary analysis with Bio3D. *BMC Bioinformatics* **15**, 399 (2014).
40. Durrant, J. D., Votapka, L., Sørensen, J. & Amaro, R. E. POVME 2.0: An enhanced tool for determining pocket shape and volume characteristics. *J. Chem. Theory Comput.* **10**, 5047–5056 (2014).
41. Schrödinger, LLC. *The {PyMOL} Molecular Graphics System, Version~1.8*. (2015).
42. Belcher, J. *et al.* Structure and biochemical properties of the alkene producing cytochrome p450 OleTJE (CYP152l1) from the jeotgalicoccus sp. 8456 bacterium. *J. Biol. Chem.* **289**, 6535–6550 (2014).
