# Supplementary material for: Pervasive cooperative mutational effects on multiple catalytic enzyme traits emerge via long-range conformational dynamics
Source: Nat Commun. 2021 Mar 12;12:1621. doi: 10.1038/s41467-021-21833-w (PMC7955134; doi:10.1038/s41467-021-21833-w)
Supplement: Supplementary file 3 — Description of Additional Supplementary Files [file 41467_2021_21833_MOESM3_ESM.pdf]

Description of Additional Supplementary Files

Supplementary Movie 1: Rotation of 1 in the active site of III mutant. At first, testosterone (1) is bound in pose 15, perpendicularly with respect the haem plane and interacting with T260 and A330 through its hydroxyl and carbonyl moieties, respectively. Gradually, 1 assumes a lying pose over its α-side, and the subsequent (1)CO-T327 and (1)OH-A87 interactions start driving a counterclockwise rotation of 1 inside the haem pocket. Afterwards, (1)CO-G265 followed by (1)OH-T327 interactions complete the 180° rotation of 1, which further on assumes its final binding pose 2 perpendicular to the haem plane.

Supplementary Movie 2: Binding trajectory of 1 in I-- mutant using accelerated MD simulations. Fetching of (1) takes places through the F-G loop / β1 hairpin. The substrate enters in the enzyme pocket from the access channel 2a and there it reorients until a network of coupled conformational changes that allows the path of (1) towards the active site occur simultaneously. The G helix adopts a bend conformation, which impacts F helix and β1 sheet conformation, and in turn shifts B' helix and retreats β4 sheet, enabling 1 progression to the catalytic centre.
