# Supplementary material for: Pervasive cooperative mutational effects on multiple catalytic enzyme traits emerge via long-range conformational dynamics
Source: Nat Commun. 2021 Mar 12;12:1621. doi: 10.1038/s41467-021-21833-w (PMC7955134; doi:10.1038/s41467-021-21833-w)
Supplement: Supplementary file 6 — Reporting Summary [file 41467_2021_21833_MOESM6_ESM.pdf]

nature research

Corresponding author(s): Manfred T. Reetz & Silvia Osuna

Last updated by author(s): Jan 13, 2021

Reporting Summary

Nature Research wishes to improve the reproducibility of the work that we publish. This form provides structure for consistency and transparency in reporting. For further information on Nature Research policies, see our Editorial Policies and the Editorial Policy Checklist.

Statistics

For all statistical analyses, confirm that the following items are present in the figure legend, table legend, main text, or Methods section.

n/a	Confirmed	
☐	☒	The exact sample size (*n*) for each experimental group/condition, given as a discrete number and unit of measurement
☐	☒	A statement on whether measurements were taken from distinct samples or whether the same sample was measured repeatedly
☒	☐	The statistical test(s) used AND whether they are one- or two-sided<br>*Only common tests should be described solely by name; describe more complex techniques in the Methods section.*
☒	☐	A description of all covariates tested
☒	☐	A description of any assumptions or corrections, such as tests of normality and adjustment for multiple comparisons
☐	☒	A full description of the statistical parameters including central tendency (e.g. means) or other basic estimates (e.g. regression coefficient) AND variation (e.g. standard deviation) or associated estimates of uncertainty (e.g. confidence intervals)
☒	☐	For null hypothesis testing, the test statistic (e.g. *F*, *t*, *r*) with confidence intervals, effect sizes, degrees of freedom and *P* value noted<br>*Give P values as exact values whenever suitable.*
☒	☐	For Bayesian analysis, information on the choice of priors and Markov chain Monte Carlo settings
☒	☐	For hierarchical and complex designs, identification of the appropriate level for tests and full reporting of outcomes
☒	☐	Estimates of effect sizes (e.g. Cohen's *d*, Pearson's *r*), indicating how they were calculated

Our web collection on statistics for biologists contains articles on many of the points above.

Software and code

Policy information about availability of computer code

Data collection	HPLC data acquisition was collected by Shimadzu LCsolution software version 3 in a LC-2010 HT Shimadzu system. Kinetic data was collected using the UV-Vis-NIR Spectra Manager software 2 in JASCO V-650 spectrophotometer. Data resulting from Quantum Mechanics (QM) was collected using Gaussian09 (rev D.01) software in a in-house CPU cluster (node used composed by Intel Xeon E5-2670 CPUs). Data resulting from Molecular Dynamics (MD) was collected using Amber16 software with ff99SBildn force field in a in-house GPU cluster (composed by Nvidia GTX1080 GPUs). P450-BM3 mutant structures used for MD were collected using the RosettaBackrub web-server and the protonation state obtained with the H++ web-server.
Data analysis	Data were analyzed using Microsoft Excel 365 MSO version 2012 (16.0.13530.20054) 32-bit, GraphPad Prism version 9 for the graphs, Surfer version 8 for the fitness pathway landscapes and Python version 3.7 for writing the epistasis code, which is deposited as https://github.com/matteoferla/Epistasis_Calculator. For DNA analysis, the freeware ApE plasmid editor version 2.0.44 (by Wayne Davis) was used as well as MegAlign from DNASTAR Lasergene version 11. Data resulting from MD simulations were analyzed using PyEMMA v2.4 for the conformational population analysis, Bio3D package (R v3.3.2) for the Principal Component Analysis (PCA), POVME v2.0 for volume mapping, Python v2.7 and Seaborn library v0.9.0 for the Kernel Density Estimation (KDE) plots, DynaComm (written in Python v2.7 by S. Osuna) for the Shortest Path Map (SPM), CPPTRAJ (available in Amber16 package) for (i) monitoring distance and angle used in KDE, (ii) obtaining correlation and proximity matrix used in SPM (iii) MD trajectory clustering for the conformational population analysis. For exploration of protein structure, PyMol Molecular Graphics System, V 1.5.0.4 was used. For the exploration of molecular structure, Visual Molecular Dynamics v1.9.3 was used.

For manuscripts utilizing custom algorithms or software that are central to the research but not yet described in published literature, software must be made available to editors and reviewers. We strongly encourage code deposition in a community repository (e.g. GitHub). See the Nature Research guidelines for submitting code & software for further information.

Data

Policy information about availability of data

All manuscripts must include a data availability statement. This statement should provide the following information, where applicable:

- Accession codes, unique identifiers, or web links for publicly available datasets
- A list of figures that have associated raw data
- A description of any restrictions on data availability

The authors declare that all data supporting the findings of this study are available within the paper and its supplementary information files. Source data are provided with this paper. Computer code for determination of epistatic effects is available at DOI: 10.5281/zenodo.4423157. The Dynacomm computer code for the SPM (doi: 10.1021/acscatal.7b02954) is available from the author upon reasonable request. PDB ID: 1FAG has been referenced in the manuscript.

Field-specific reporting

Please select the one below that is the best fit for your research. If you are not sure, read the appropriate sections before making your selection.

[x] Life sciences [ ] Behavioural & social sciences [ ] Ecological, evolutionary & environmental sciences

For a reference copy of the document with all sections, see nature.com/documents/nr-reporting-summary-flat.pdf

Life sciences study design

All studies must disclose on these points even when the disclosure is negative.

Sample size	No statistical methods were used to predetermine sample size. Sample sizes were determined by considering biological and technical variability from previous experiments.
Data exclusions	No data was excluded from the analysis.
Replication	At least 2 or 3 independent replicates were done.
Randomization	Randomization was not applicable to the experiments done since no statistical comparisons were made.
Blinding	Binding was not relevant since no statistical comparisons were made.

Reporting for specific materials, systems and methods

We require information from authors about some types of materials, experimental systems and methods used in many studies. Here, indicate whether each material, system or method listed is relevant to your study. If you are not sure if a list item applies to your research, read the appropriate section before selecting a response.

Materials & experimental systems

n/a	Involved in the study	
[x]	[ ]	Antibodies
[x]	[ ]	Eukaryotic cell lines
[x]	[ ]	Palaeontology and archaeology
[x]	[ ]	Animals and other organisms
[x]	[ ]	Human research participants
[x]	[ ]	Clinical data
[x]	[ ]	Dual use research of concern

Methods

n/a	Involved in the study	
[x]	[ ]	ChIP-seq
[x]	[ ]	Flow cytometry
[x]	[ ]	MRI-based neuroimaging
